# Correction: Including population and environmental dynamic heterogeneities in continuum models of collective behaviour with applications to locust foraging and group structure

**DOI:** 10.1371/journal.pcbi.1013167

**Published:** 2025-06-09

**Authors:** The PLOS Computational Biology Staff

**Notice of Republication**

This article was republished on May 23, 2025, to correct errors in references 6 and 21 that were introduced during the typesetting process. The publisher apologizes for these errors. Please download the article again to view the corrected version. Both the originally published, uncorrected article and the republished, corrected article are provided here for reference.

## Supporting information

S1 File Originally published, uncorrected article. (PDF)

S2 File Republished, corrected article. (PDF)
