# Supplementary material for: Including population and environmental dynamic heterogeneities in continuum models of collective behaviour with applications to locust foraging and group structure
Source: PLoS Comput Biol. 2025 Apr 15;21(4):e1011469. doi: 10.1371/journal.pcbi.1011469 (PMC11999712; doi:10.1371/journal.pcbi.1011469)
Supplement: S1 Appendix — The full detailed derivation of the flux terms given in the model section. (PDF) [file pcbi.1011469.s001.pdf]

# S1 Appendix: Model Derivation

Fillipe Georgiou[1], Camille Buhl[2], J.E.F. Green[3],
Bishnu Lamichhane[4] and Ngamta Thamwattana[4]
[1] Institute for Mathematical Innovation, University of Bath,
Bath, United Kingdom.
[2] School of Agriculture, Food and Wine, University of Adelaide,
Adelaide, Australia.
[3] School of Computer & Mathematical Sciences, University of Adelaide,
Adelaide, Australia.
[4] School of Information and Physical Sciences, University of Newcastle,
Callaghan, Australia.

February 24, 2025

## 1 Model Derivation

We begin by presenting a continuous kinematic model of collective behaviour that includes both local and non-local inter-individual interactions, as well as environmental interactions, with all interactions mediated by the internal state of the organism. For simplicity, we consider a single population, which is represented as a density of individuals (number per unit area), $\rho$, at point $\boldsymbol{x}$ in space, at time $t$, and with internal state $\boldsymbol{n}$. Here, $\boldsymbol{n} = (n_1, .., n_N)$ is an $N$-dimensional continuous variable, where each state $n_i$ $(i = 1, \ldots, N)$ can take a value on the interval $[0, 1]$. The states can be used to represent a range of characteristics of individual organisms, such as their degree of satiation, age, stage of gregarisation, *etc.*, as a fraction of the greatest possible value, *i.e.*, all the states are normalised and dimensionless. We will term the combination of all possible internal states the state space, $\Omega_{\boldsymbol{n}}$ (*i.e.*, the state space is an $N$-dimensional hypercube).

We make the following modelling assumptions:

1. Organisms can be classified by their internal state and this state is continuous in nature.
2. Environmental interactions are local in nature (*i.e.,* an organism at point $\boldsymbol{x}$ is only influenced by the environment at point $\boldsymbol{x}$) .
3. Local interactions that directly affect movement between organisms are repulsive (*i.e.,* individuals try to avoid collisions or close physical contact).
4. Organisms also experience a non-local (*i.e.,* longer-ranged) interaction (this could include both attractive or repulsive interactions mediated by longer-range sensing such as sight, hearing or smell).
5. The nature of all interactions depends upon the organism's internal state and local environmental conditions, and not by those of other organisms.

In this model organisms are represented as a density of individuals (number per unit area) at point $\boldsymbol{x}$ in space, at time $t$, and with a $N$-dimensional continuous internal state, $\boldsymbol{n} = (n_1, .., n_N)$, where each state can take a value on the interval $[0, 1]$. We will term the combination of all possible internal states as the state space (i.e. the state space is an $N$-dimensional hypercube). This state may characterise their satiation, age, stage of gregarisation, etc. as a fraction of the greatest possible value, i.e. all the states are normalised and

unit-less. Let the density of organisms in space, state, and time be given by, $\rho(\boldsymbol{x}, \boldsymbol{n}, t)$ with the total local density in space defined as

$$\bar{\rho}(\boldsymbol{x}, t) = \int_{\Omega_{\boldsymbol{n}}} \rho(\boldsymbol{x}, \boldsymbol{n}, t) d\boldsymbol{n}, \tag{1}$$

where $\Omega_{\boldsymbol{n}}$ is our complete state domain, we will also define $\Omega_{\boldsymbol{x}}$ as our spatial domain. For later convenience we will also define the total mass of organisms as

$$M = \int_{\Omega_{\boldsymbol{x}}} \bar{\rho}(\boldsymbol{x}, t)\, d\boldsymbol{x}, \tag{2}$$

as well as a generic term for the environment, $E$, that might represent such environmental conditions as food density, sunlight, temperature, etc. $E$ may be a vector of values that is constant, or change in space and time, or due to interactions with organisms, etc.

We assume that the time-scales we are investigating are shorter than the life cycle of the organism, ignoring births and deaths and thus conserving the total number of organisms. We consider local organism-organism interactions (e.g. crowding), local organism-environment interactions (e.g. foraging), and non-local organism-organism interactions (e.g. sight and smell mediated interactions between individuals). We also allow for a movement within the state space (e.g. the change in an organisms hunger level). Hence, conservation laws give equations of the form

$$\frac{\partial \rho}{\partial t} + \nabla \cdot (\boldsymbol{J}_{\text{local}} + \boldsymbol{J}_{\text{non-local}}) + \nabla_{\boldsymbol{n}} \cdot (\boldsymbol{J}_{\text{state}}) \;= 0, \tag{3}$$

where $\boldsymbol{J}_{\text{local}}$ is the flux due to local interactions, $\boldsymbol{J}_{\text{non-local}}$ is the flux due to non-local interactions, $\boldsymbol{J}_{\text{state}}$ is the flux around the state space and $\nabla_{\boldsymbol{n}}$ represents the differential operator, $\nabla$, applied to the state space variables, i.e.

$$\nabla_{\boldsymbol{n}} = \left( \frac{\partial}{\partial n_1}, ..., \frac{\partial}{\partial n_N} \right).$$

## 1.1 Derivation of local-flux

Following the work of Painter and Sherratt [4], we derive the local interactions as the limit of a lattice model,

We begin here by considering organism movement on a $(N+1)$-dimensional lattice with one dimension of space and $N$ dimensions corresponding to the organisms internal state with random movement restricted to the spatial dimension and the state space traversed by some advective velocity. Let each dimension, $d$, of the internal state have $m_d$ possible choices, with an arbitrary state at the indexed location, $\boldsymbol{j}$, given by the $N$ dimensional vector $\boldsymbol{n_j}$. Let $\rho_{i,\boldsymbol{j}}^t$ be the number of organisms at site $i$ and with internal state $\boldsymbol{n_j}$, at time $t$, and $E_i^t$ be the environmental conditions at site $i$. In addition let,

$$\bar{\rho}_i^t = \sum_{\boldsymbol{j} \in \Omega_n} \rho_{i,\boldsymbol{j}}^t, \tag{4}$$

i.e. $\bar{\rho}_i^t$ is the total number of organisms at spatial site $i$ and time $t$ regardless of state.

We assume that the transition probabilities for an organism at the $(i, \boldsymbol{j})^{th}$ site depends on the environmental conditions at that site, the state of the organism, and the relative population density between the current site and neighbouring sites. If we let $\mathcal{T}_{i,\boldsymbol{j}}^{\pm}$ be the probability at which organisms at site $(i, \boldsymbol{j})$ move to the right, $+$, and left, $-$, during a timestep, then our transition probabilities are

$$\mathcal{T}_{i,\boldsymbol{j}}^{\pm} = F(\boldsymbol{n_j}, E_i)(\alpha + \beta(\tau(\bar{\rho}_i) - \tau(\bar{\rho}_{i\pm1}))), \tag{5}$$

where $F$ is a function of environmental conditions and state, $\tau$ is a function related to the local organism density, and $\alpha$ and $\beta$ are constants. Finally, individuals transition around the state-space given by the boundary fluxes

$$S_{i,\boldsymbol{j}} = \sum_{k=1}^{N} J_{i,\boldsymbol{j}-\frac{1}{2}\boldsymbol{e}_k} - J_{i,\boldsymbol{j}+\frac{1}{2}\boldsymbol{e}_k}, \tag{6}$$

where $\boldsymbol{e}_k$ is the unit state-space vector in the arbitrary direction $k$. We also assume that individuals either move in space or change internal state at each time step. Then the number of individuals at site $(i, \boldsymbol{j})$ at time $t + \Delta t$ is given by

$$\rho_{i,\boldsymbol{j}}^{t+\Delta t} = \rho_{i,\boldsymbol{j}}^{t} + \mathcal{T}_{i+1,\boldsymbol{j}}^{-}\rho_{i+1,\boldsymbol{j}}^{t} + \mathcal{T}_{i-1,\boldsymbol{j}}^{+}\rho_{i-1,\boldsymbol{j}}^{t} - (\mathcal{T}_{i,\boldsymbol{j}}^{-} + \mathcal{T}_{i,\boldsymbol{j}}^{+})\rho_{i,\boldsymbol{j}}^{t} + S_{i,\boldsymbol{j}}. \tag{7}$$

Substituting (5) and (6) into (7) gives

$$\begin{aligned}\rho_{i,\boldsymbol{j}}^{t+\Delta t} =& \rho_{i,\boldsymbol{j}}^{t} + F(\boldsymbol{n_j}, E_{i+1})(\alpha + \beta(\tau(\bar{\rho}_{i+1}) - \tau(\bar{\rho}_i)))\rho_{i+1,\boldsymbol{j}}^{t} \\ & + F(\boldsymbol{n_j}, E_{i-1})(\alpha + \beta(\tau(\bar{\rho}_{i-1}) - \tau(\bar{\rho}_i)))\rho_{i-1,\boldsymbol{j}}^{t} \\ & - [F(\boldsymbol{n_j}, E_i)(\alpha + \beta(\tau(\bar{\rho}_i) - \tau(\bar{\rho}_{i-1}))) \\ & + F(\boldsymbol{n_j}, E_i)(\alpha + \beta(\tau(\bar{\rho}_i) - \tau(\bar{\rho}_{i+1})))]\rho_{i,\boldsymbol{j}}^{t} \\ & + \sum_{k=1}^{N} J_{i,\boldsymbol{j}-\frac{1}{2}\boldsymbol{e}_k} - J_{i,\boldsymbol{j}+\frac{1}{2}\boldsymbol{e}_k}.\end{aligned} \tag{8}$$

We the rearrange (8) to take out the common factors $\alpha$ and $\beta$, giving

$$\begin{aligned}\rho_{i,\boldsymbol{j}}^{t+\Delta t} =& \rho_{i,\boldsymbol{j}}^{t} + \alpha[F(\boldsymbol{n_j}, E_{i+1})\rho_{i+1,\boldsymbol{j}}^{t} + F(\boldsymbol{n_j}, E_{i-1})\rho_{i-1,\boldsymbol{j}}^{t} - 2F(\boldsymbol{n_j}, E_i)\rho_{i,\boldsymbol{j}}^{t}] \\ & + \beta\left[F(\boldsymbol{n_j}, E_{i+1,\boldsymbol{j}})\rho_{i+1,\boldsymbol{j}}^{t}(\tau(\bar{\rho}_{i+1}) - \tau(\bar{\rho}_i))\right. \\ & + F(c_{\boldsymbol{n_j},i-1})\rho_{i-1,\boldsymbol{j}}^{t}(\tau(\bar{\rho}_{i-1}) - \tau(\bar{\rho}_i)) \\ & \left. - F(\boldsymbol{n_j}, E_i)\rho_{i,\boldsymbol{j}}^{t}(2\tau(\bar{\rho}_i) - \tau(\bar{\rho}_{i-1}) - \tau(\bar{\rho}_{i+1}))\right] \\ & + \sum_{k=1}^{N} J_{i,\boldsymbol{j}-\frac{1}{2}\boldsymbol{e}_k} - J_{i,\boldsymbol{j}+\frac{1}{2}\boldsymbol{e}_k}.\end{aligned} \tag{9}$$

We then Taylor expand the terms in (9) to obtain the equation in relation to the site $(i, \boldsymbol{j})$ at time $t$ only. Beginning with,

$$\rho_{i,\boldsymbol{j}}^{t+\Delta t} = \rho_{i,\boldsymbol{j}}^{t} + \Delta t \frac{\partial \rho_{i,\boldsymbol{j}}^{t}}{\partial t} + \mathcal{O}(\Delta t^2). \tag{10}$$

Then for the terms related to $\alpha$ we get

$$\begin{aligned}\alpha[\cdot] =& \alpha\left[F(\boldsymbol{n_j}, E_i)\rho_{i,\boldsymbol{j}}^{t} + \Delta x \frac{\partial}{\partial x}(F(\boldsymbol{n_j}, E_i)\rho_{i,\boldsymbol{j}}^{t}) + \frac{\Delta x^2}{2}\frac{\partial^2}{\partial x^2}(F(\boldsymbol{n_j}, E_i)\rho_{i,\boldsymbol{j}}^{t}) + \frac{\Delta x^3}{6}\frac{\partial^3}{\partial x^3}(F(\boldsymbol{n_j}, E_i)\rho_{i,\boldsymbol{j}}^{t})\right. \\ & F(\boldsymbol{n_j}, E_i)\rho_{i,\boldsymbol{j}}^{t} - \Delta x \frac{\partial}{\partial x}(F(\boldsymbol{n_j}, E_i)\rho_{i,\boldsymbol{j}}^{t}) + \frac{\Delta x^2}{2}\frac{\partial^2}{\partial x^2}(F(\boldsymbol{n_j}, E_i)\rho_{i,\boldsymbol{j}}^{t}) - \frac{\Delta x^3}{6}\frac{\partial^3}{\partial x^3}(F(\boldsymbol{n_j}, E_i)\rho_{i,\boldsymbol{j}}^{t}) \\ & \left. -2F(\boldsymbol{n_j}, E_i)\rho_{i,\boldsymbol{j}}^{t} + \mathcal{O}(\Delta x^4)\right], \\ =& \alpha \Delta x^2 \frac{\partial^2}{\partial x^2}(F(\boldsymbol{n_j}, E_i)\rho_{i,\boldsymbol{j}}^{t}) + \mathcal{O}(\Delta x^4),\end{aligned} \tag{11}$$

as the $0^{th}, 1^{st}$, and $, 3^{rd}$ order terms of $\Delta x$ cancel each other out. We then turn our attention to our terms involving $\beta$, we will Taylor expand each multiplication individually as otherwise the terms become

unimaginably unmanageable. To begin,

$$
\begin{aligned}
\mathcal{R} =& F(\boldsymbol{n_j}, E_{i+1})\rho^t_{i+1,\boldsymbol{j}}(\tau(\bar{\rho}_{i+1}) - \tau(\bar{\rho}_i)) \\
=& \left[F(\boldsymbol{n_j}, E_i)\rho^t_{i,\boldsymbol{j}} + \Delta x \frac{\partial}{\partial x}(F(\boldsymbol{n_j}, E_i)\rho^t_{i,\boldsymbol{j}}) + \frac{\Delta x^2}{2}\frac{\partial^2}{\partial x^2}(F(\boldsymbol{n_j}, E_i)\rho^t_{i,\boldsymbol{j}}) + \frac{\Delta x^3}{6}\frac{\partial^3}{\partial x^3}(F(\boldsymbol{n_j}, E_i)\rho^t_{i,\boldsymbol{j}})\right] \\
& \cdot \left[\tau(\bar{\rho}_i) - \tau(\bar{\rho}_i) + \Delta x \frac{\partial}{\partial x}(\tau(\bar{\rho}_i)) + \frac{\Delta x^2}{2}\frac{\partial^2}{\partial x^2}(\tau(\bar{\rho}_i)) + \frac{\Delta x^3}{6}\frac{\partial^3}{\partial x^3}(\tau(\bar{\rho}_i))\right] + \mathcal{O}(\Delta x^4) \\
=& F(\boldsymbol{n_j}, E_i)\rho^t_{i,\boldsymbol{j}} \left[\Delta x \frac{\partial}{\partial x}\left(\tau(\bar{\rho}_i)\right) + \frac{\Delta x^2}{2}\frac{\partial^2}{\partial x^2}\left(\tau(\bar{\rho}_i)\right) + \frac{\Delta x^3}{6}\frac{\partial^3}{\partial x^3}\left(\tau(\bar{\rho}_i)\right)\right] \\
& + \Delta x \frac{\partial}{\partial x}(F(\boldsymbol{n_j}, E_i)\rho^t_{i,\boldsymbol{j}}) \left[\Delta x \frac{\partial}{\partial x}(\tau(\bar{\rho}_i)) + \frac{\Delta x^2}{2}\frac{\partial^2}{\partial x^2}(\tau(\bar{\rho}_i))\right] \\
& + \frac{\Delta x^2}{2}\frac{\partial^2}{\partial x^2}\left(F(\boldsymbol{n_j}, E_i)\rho^t_{i,\boldsymbol{j}}\right)\left[\Delta x \frac{\partial}{\partial x}(\tau(\bar{\rho}_i))\right] + \mathcal{O}(\Delta x^4),
\end{aligned} \tag{12}
$$

and

$$
\begin{aligned}
\mathcal{L} =& F(\boldsymbol{n_j}, E_{i-1})\rho^t_{i-1,\boldsymbol{j}}(\tau(\bar{\rho}_{i-1}) - \tau(\bar{\rho}_i)) \\
=& \left[F(\boldsymbol{n_j}, E_i)\rho^t_{i,\boldsymbol{j}} - \Delta x \frac{\partial}{\partial x}(F(\boldsymbol{n_j}, E_i)\rho^t_{i,\boldsymbol{j}}) + \frac{\Delta x^2}{2}\frac{\partial^2}{\partial x^2}(F(\boldsymbol{n_j}, E_i)\rho^t_{i,\boldsymbol{j}}) - \frac{\Delta x^3}{6}\frac{\partial^3}{\partial x^3}(F(\boldsymbol{n_j}, E_i)\rho^t_{i,\boldsymbol{j}})\right] \\
& \cdot \left[\tau(\bar{\rho}_i) - \tau(\bar{\rho}_i) - \Delta x \frac{\partial}{\partial x}(\tau(\bar{\rho}_i)) + \frac{\Delta x^2}{2}\frac{\partial^2}{\partial x^2}(\tau(\bar{\rho}_i)) - \frac{\Delta x^3}{6}\frac{\partial^3}{\partial x^3}(\tau(\bar{\rho}_i))\right] + \mathcal{O}(\Delta x^4) \\
=& F(\boldsymbol{n_j}, E_i)\rho^t_{i,\boldsymbol{j}} \left[-\Delta x \frac{\partial}{\partial x}\left(\tau(\bar{\rho}_i)\right) + \frac{\Delta x^2}{2}\frac{\partial^2}{\partial x^2}\left(\tau(\bar{\rho}_i)\right) - \frac{\Delta x^3}{6}\frac{\partial^3}{\partial x^3}\left(\tau(\bar{\rho}_i)\right)\right] \\
& - \Delta x \frac{\partial}{\partial x}(F(\boldsymbol{n_j}, E_i)\rho^t_{i,\boldsymbol{j}}) \left[-\Delta x \frac{\partial}{\partial x}(\tau(\bar{\rho}_i)) + \frac{\Delta x^2}{2}\frac{\partial^2}{\partial x^2}(\tau(\bar{\rho}_i))\right] \\
& + \frac{\Delta x^2}{2}\frac{\partial^2}{\partial x^2}\left(F(\boldsymbol{n_j}, E_i)\rho^t_{i,\boldsymbol{j}}\right)\left[-\Delta x \frac{\partial}{\partial x}(\tau(\bar{\rho}_i))\right] + \mathcal{O}(\Delta x^4),
\end{aligned} \tag{13}
$$

and finally,

$$
\begin{aligned}
\mathcal{C} =& - F(\boldsymbol{n_j}, E_i)\rho^t_{i,\boldsymbol{j}}(2\tau(\bar{\rho}_i) - \tau(\bar{\rho}_{i-1}) - \tau(\bar{\rho}_{i+1})) \\
=& - F(\boldsymbol{n_j}, E_i)\rho^t_{i,\boldsymbol{j}} \Bigg[2\tau(\bar{\rho}_i) - \tau(\bar{\rho}_i) + \Delta x \frac{\partial}{\partial x}(\tau(\bar{\rho}_i)) - \frac{\Delta x^2}{2}\frac{\partial^2}{\partial x^2}(\tau(\bar{\rho}_i)) + \frac{\Delta x^3}{6}\frac{\partial^3}{\partial x^3}(\tau(\bar{\rho}_i)) \\
& -\tau(\bar{\rho}_i) - \Delta x \frac{\partial}{\partial x}(\tau(\bar{\rho}_i)) - \frac{\Delta x^2}{2}\frac{\partial^2}{\partial x^2}(\tau(\bar{\rho}_i)) - \frac{\Delta x^3}{6}\frac{\partial^3}{\partial x^3}(\tau(\bar{\rho}_i))\Bigg] + \mathcal{O}(\Delta x^4), \\
=& \Delta x F(\boldsymbol{n_j}, E_i)\rho^t_{i,\boldsymbol{j}} \frac{\partial^2}{\partial x^2}(\tau(\bar{\rho}_i)) + \mathcal{O}(\Delta x^4).
\end{aligned} \tag{14}
$$

Adding (12), (13), and (14), gives

$$
\begin{aligned}
\mathcal{L} + \mathcal{C} + \mathcal{R} =& 2\Delta x^2 \left[\Delta x F(\boldsymbol{n_j}, E_i)\rho^t_{i,\boldsymbol{j}} \frac{\partial^2}{\partial x^2}(\tau(\bar{\rho}_i)) + \frac{\partial}{\partial x}(F(\boldsymbol{n_j}, E_i)\rho^t_{i,\boldsymbol{j}})\frac{\partial}{\partial x}\left(\tau(\bar{\rho}_i)\right)\right] + \mathcal{O}(\Delta x^4), \\
=& 2\Delta x^2 \frac{\partial}{\partial x}\left(F(\boldsymbol{n_j}, E_i)\rho^t_i \frac{\partial}{\partial x}\left(\tau(\bar{\rho}_i)\right)\right) + \mathcal{O}(\Delta x^4).
\end{aligned} \tag{15}
$$

Lastly, for our flux around the state-space we get

$$\sum_{k=1}^{N} J_{i,\boldsymbol{j}-\frac{1}{2}\boldsymbol{e}_k} - J_{i,\boldsymbol{j}+\frac{1}{2}\boldsymbol{e}_k} = \sum_{k=1}^{N} J_{i,\boldsymbol{j}} - \frac{\Delta n}{2}\frac{\partial J_{i,\boldsymbol{j}}}{\partial n_k} + \frac{\Delta n^2}{8}\frac{\partial^2 J_{i,\boldsymbol{j}}}{\partial n_k^2} - \left(J_{i,\boldsymbol{j}} + \frac{\Delta n}{2}\frac{\partial J_{i,\boldsymbol{j}}}{\partial n_k} + \frac{\Delta n^2}{8}\frac{\partial^2 J_{i,\boldsymbol{j}}}{\partial n_k^2}\right) + \mathcal{O}(\Delta n^3),$$
$$= \sum_{k=1}^{N} -\Delta n \frac{\partial J_{i,\boldsymbol{j}}}{\partial n_k} + \mathcal{O}(\Delta n^3). \tag{16}$$

Combining (10), (11), (15), and (16) into (9), gives,

$$\begin{aligned}\rho^t_{i,\boldsymbol{j}} + \Delta t \frac{\partial \rho^t_{i,\boldsymbol{j}}}{\partial t} + \mathcal{O}(\Delta t^2) =& \rho^t_{i,\boldsymbol{j}} + \alpha \Delta x^2 \frac{\partial^2}{\partial x^2}(F(\boldsymbol{n_j}, E_i)\rho^t_{i,\boldsymbol{j}}) \\ &+ 2\beta\Delta x^2 \frac{\partial}{\partial x}\left(F(\boldsymbol{n_j}, E_i)\rho^t_{i,\boldsymbol{j}} \frac{\partial}{\partial x}(\tau(\bar{\rho}_i))\right) \\ &- \sum_{k=1}^{N} \Delta n \frac{\partial J_{i,\boldsymbol{j}}}{\partial n_k} + \mathcal{O}(\Delta n^3) + \mathcal{O}(\Delta x^4),\end{aligned}$$

which we rearranging to obtain

$$\begin{aligned}\frac{\partial \rho^t_{i,\boldsymbol{j}}}{\partial t} =& \alpha \frac{\Delta x^2}{\Delta t}\frac{\partial^2}{\partial x^2}(F(\boldsymbol{n_j}, E_i)\rho^t_i) + 2\beta \frac{\Delta x^2}{\Delta t}\frac{\partial}{\partial x}\left(F(\boldsymbol{n_j}, E_i)\rho^t_{i,\boldsymbol{j}}\frac{\partial}{\partial x}(\tau(\bar{\rho}_i))\right) \\ &- \sum_{k=1}^{N} \frac{\Delta n}{\Delta t}\frac{\partial J_{i,\boldsymbol{j}}}{\partial n_k} + \mathcal{O}(\Delta n^3) + \mathcal{O}(\Delta x^4) + \mathcal{O}(\Delta t^2).\end{aligned} \tag{17}$$

We then substitute our function,

$$F(\boldsymbol{n_j}, E_i) = f_l(\boldsymbol{n_j}, E_i), \tag{18}$$

to obtain

$$\begin{aligned}\frac{\partial \rho^t_i}{\partial t} =& \alpha \frac{\Delta x^2}{\Delta t}\frac{\partial^2}{\partial x^2}\left(f_l(\boldsymbol{n_j}, E_i)\rho^t_{i,\boldsymbol{j}}\right) + 2\beta \frac{\Delta x^2}{\Delta t}\frac{\partial}{\partial x}\left(f_l(\boldsymbol{n_j}, E_i)\rho^t_{i,\boldsymbol{j}}\frac{\partial}{\partial x}\left(\tau(\bar{\rho}_i) = \bar{\rho}_i^2\right)\right) \\ &- \sum_{k=1}^{N} \frac{\Delta n}{\Delta t}\frac{\partial J_{i,\boldsymbol{j}}}{\partial n_k} + \mathcal{O}(\Delta n^3) + \mathcal{O}(\Delta x^4) + \mathcal{O}(\Delta t^2).\end{aligned} \tag{19}$$

We then take the limit as $\Delta x, \Delta t, \Delta n \to 0$ such that,

$$\lim_{\substack{\Delta x\to 0\\ \Delta t \to 0}} \alpha\frac{\Delta x^2}{\Delta t} = D, \text{ and } \lim_{\substack{\Delta x\to 0\\ \Delta t \to 0}} 2\beta\frac{\Delta x^2}{\Delta t} = D\gamma, \text{ and } \lim_{\substack{\Delta n\to 0\\ \Delta t \to 0}} \frac{\Delta n}{\Delta t} = 1, \tag{20}$$

to find,

$$\frac{\partial \rho}{\partial t} = D\frac{\partial^2}{\partial x^2}(f_l(\boldsymbol{n}, E)\rho) + D\gamma\frac{\partial}{\partial x}\left(f_l(\boldsymbol{n}, E)\rho\frac{\partial}{\partial x}(\tau(\bar{\rho}_i))\right) - \nabla_{\boldsymbol{n}} \cdot (\boldsymbol{J}_{\text{state}}). \tag{21}$$

Which we then rearrange to find our local flux as

$$J_{\text{local}} = -D\left[\frac{\partial}{\partial x}(f_l(\boldsymbol{n}, E)\rho) + \gamma\rho\left(f_l(\boldsymbol{n}, E)\frac{\partial}{\partial x}(\tau(\bar{\rho}_i))\right)\right]. \tag{22}$$

We note that the derivation into higher dimensions follows the same process but the equations become even more unwieldy and is therefore omitted here. We thus write,

$$J_{\text{local}} = -D\left[\nabla(f_l(\boldsymbol{n}, E)\rho) + \gamma\rho\left(f_l(\boldsymbol{n}, E)\nabla(\tau(\bar{\rho}_i))\right)\right]. \tag{23}$$

## 1.2 Derivation of non-local-flux

Next, for our non-local interactions, we adopt the fluxes used originally by Mogilner and Edelstein-Keshet [3], and in numerous subsequent studies [2, 5, 6]. Here we give a heuristic derivation following the work of Bodnar and Velazquez [1]. To begin, we give a kinematic SPP model in which organisms interact with organisms in any state and with strength proportional to their own internal state and local environmental conditions,

$$\dot{X}_i = -\frac{1}{N} f_n(X_i, E) \sum_{j=1}^{N} \nabla_x Q(X_i - X_j), \tag{24}$$

where $Q$ is some social potential relating the distance between organisms to the strength of interaction, and $F_n(X_i, E)$ translates the organisms state and local environment into a strength of movement.

We then divide up the space into small intervals of space and state, with the number of organisms in and interval, $y$, with state, $\boldsymbol{n}$, given by,

$$\rho(y, n, t) = \frac{\#\{X_i \in [y - h, y + h, \boldsymbol{n} - \epsilon, \boldsymbol{n} + \epsilon]\}}{2h + 2^N \epsilon}, \tag{25}$$

with the total number of individuals in an interval of space given by

$$\bar{\rho}(y, t) = \frac{\#\{X_i \in [y - h, y + h]\}}{2h}, \tag{26}$$

where in the continuum limit under consideration, $h$ satisfies

$$\delta \ll h \ll 1, \tag{27}$$

with $\delta$ being the average distance between individuals in space. We then note by assuming that in each interval the individuals are locally at equilibrium then as $h \to 0, \epsilon \to 0$ the velocity of an individual depends only on its location and state, i.e.

$$v(x, \boldsymbol{n}, t) = -f_n(\boldsymbol{n}, E) \nabla \sum_{n=1}^{N} \left[ Q(x - X_n) \right]. \tag{28}$$

This allows us to approximate the velocity field using integrals to give

$$v(x, \boldsymbol{n}, t) = -f_n(\boldsymbol{n}, E) \nabla \int Q(x - y) \bar{\rho}(y, t) dy. \tag{29}$$

The evolution of the macroscopic density as $\delta, h, \epsilon \to 0$ is then given by the continuity equation

$$\frac{\partial \rho(x, \boldsymbol{n}, t)}{\partial t} + \frac{\partial j}{\partial x} = 0, \tag{30}$$

with particle flux given by

$$j = \rho v. \tag{31}$$

Thus, our non-local flux is

$$\boldsymbol{J}_{\text{non-local}} = -f_n(\boldsymbol{n}, E) \nabla (Q(\boldsymbol{x}) * \bar{\rho}(\boldsymbol{x}, t)) \rho(\boldsymbol{x}, \boldsymbol{n}, t), \tag{32}$$

where $*$ is the convolution operation, $Q(\boldsymbol{x})$ is the social potential, and $f_n(\boldsymbol{n}, E)$ is a function relating internal state and environmental conditions to the strength and direction of the non-local force.

### 1.3 State-flux

Finally, we assume that organisms change their internal state based on some $N$ dimensional state vector field (akin to a velocity vector), $\boldsymbol{v}_n$ giving our flux as

$$\boldsymbol{J}_{\text{state}} = \boldsymbol{v}_n \rho, \tag{33}$$

with each element of $\boldsymbol{v}_n = (\dot{n_1}, ..., \dot{n_N})$ being a function describing the rate of change in state.

### 1.4 Full model

Combining (23), (32), and (33) into (3) gives,

$$\frac{\partial \rho}{\partial t} + \nabla \cdot (\boldsymbol{v}_x \rho) + \nabla_{\boldsymbol{n}} \cdot (\boldsymbol{v}_n \rho) = D \nabla \cdot \left[ \nabla \left( f_l(\boldsymbol{n}, E) \rho \right) \right], \tag{34}$$

with

$$\boldsymbol{v}_x = -f_n(\boldsymbol{n}, E) \nabla (Q * \bar{\rho}) - D \left[ \gamma f_l(\boldsymbol{n}, E) \nabla \left( \tau(\bar{\rho}) \right) \right]. \tag{35}$$

We then chose to rewrite this as an advection-diffusion equation separating out $f_l(\boldsymbol{n}, E)$ from $\nabla \left( f_l(\boldsymbol{n}, E) \rho \right)$ using

$$\nabla \left( f_l(\boldsymbol{n}, E) \rho \right) = f_l(\boldsymbol{n}, E) \nabla \rho + \rho \nabla f_l(\boldsymbol{n}, E). \tag{36}$$

This allows us to see the advective component provided by $f_l(\boldsymbol{n}, E)$ more explicitly. By doing this we get the full generalised model as

$$\frac{\partial \rho}{\partial t} + \nabla \cdot (\boldsymbol{v}_x \rho) + \nabla_{\boldsymbol{n}} \cdot (\boldsymbol{v}_n \rho) = D \nabla \cdot \left[ f_l(\boldsymbol{n}, E) \nabla \rho \right], \tag{37}$$

with

$$\boldsymbol{v}_x = -f_n(\boldsymbol{n}, E) \nabla (Q * \bar{\rho}) - D \left[ \nabla f_l(\boldsymbol{n}, E) + \gamma f_l(\boldsymbol{n}, E) \nabla \left( \tau(\bar{\rho}) \right) \right]. \tag{38}$$

## References


[1] M. Bodnar and J. J. L. Velazquez. “An integro-differential equation arising as a limit of individual cell-based models”. In: ***Journal of Differential Equations*** 222.2 (Mar. 2006), pp. 341–380. ISSN: 0022-0396. DOI: 10.1016/j.jde.2005.07.025.

[2] Fillipe Georgiou et al. “Modelling locust foraging: How and why food affects group formation”. In: ***PLOS Computational Biology*** 17.7 (July 2021), e1008353. ISSN: 1553-7358. DOI: 10.1371/journal.pcbi.1008353.

[3] A. Mogilner and L. Edelstein-Keshet. “A non-local model for a swarm”. In: ***Journal of Mathematical Biology*** 38.6 (June 1999), pp. 534–570. ISSN: 1432-1416. DOI: 10.1007/s002850050158.

[4] K. J. Painter and J. A. Sherratt. “Modelling the movement of interacting cell populations”. In: ***Journal of Theoretical Biology*** 225.3 (Dec. 2003), pp. 327–339. ISSN: 0022-5193. DOI: 10.1016/S0022-5193(03)00258-3.

[5] C. M. Topaz, A. L. Bertozzi, and M. A. Lewis. “A Nonlocal Continuum Model for Biological Aggregation”. In: ***Bulletin of Mathematical Biology*** 68.7 (July 2006), p. 1601. ISSN: 1522-9602. DOI: 10.1007/s11538-006-9088-6.

[6] C. M. Topaz et al. “Locust Dynamics: Behavioral Phase Change and Swarming”. In: ***PLOS Computational Biology*** 8.8 (Aug. 2012), e1002642. ISSN: 1553-7358. DOI: 10.1371/journal.pcbi.1002642.
