# Supplementary material for: Including population and environmental dynamic heterogeneities in continuum models of collective behaviour with applications to locust foraging and group structure
Source: PLoS Comput Biol. 2025 Apr 15;21(4):e1011469. doi: 10.1371/journal.pcbi.1011469 (PMC11999712; doi:10.1371/journal.pcbi.1011469)
Supplement: S2 Appendix — The full detailed derivations of the analytic results given in the PDE model analysis section for the more general model. (PDF) [file pcbi.1011469.s002.pdf]

S2 Appendix: Full analytic results

Fillipe Georgiou[1], Camille Buhl[2], J.E.F. Green[3],
Bishnu Lamichhane[4] and Ngamta Thamwattana[4]
[1] Institute for Mathematical Innovation, University of Bath,
Bath, United Kingdom.
[2] School of Agriculture, Food and Wine, University of Adelaide,
Adelaide, Australia.
[3] School of Computer & Mathematical Sciences, University of Adelaide,
Adelaide, Australia.
[4] School of Information and Physical Sciences, University of Newcastle,
Callaghan, Australia.

February 24, 2025

For this analysis our full equation is given by:

$$\frac{\partial \rho}{\partial t} + \nabla \cdot (\boldsymbol{v}_x \rho) + \nabla_{\boldsymbol{n}} \cdot (\boldsymbol{v}_n \rho) = D\nabla \cdot \left[f_l(\boldsymbol{n}, E) \nabla \rho \right], \tag{1}$$

with

$$\boldsymbol{v}_x = -f_n(\boldsymbol{n}, E)\nabla(Q * \bar{\rho}) - D\left[\nabla f_l(\boldsymbol{n}, E) + \gamma f_l(\boldsymbol{n}, E)\nabla\left(\tau(\bar{\rho})\right)\right], \tag{2}$$

and

$$\tau(\bar{\rho}) = \bar{\rho}^2. \tag{3}$$

For later convenience we will also define the total mass of organisms at time t as:

$$M(t) = \int_{\Omega_{\boldsymbol{x}}} \bar{\rho}(\boldsymbol{x}, t)\, d\boldsymbol{x}. \tag{4}$$

1 Full analytic results

In this appendix we present the calculations for our analytic results.

1.1 Density of aggregations

In order to facilitate analysis, we begin by introducing some simplifying assumptions. Under which we can estimate the maximum density and width of a aggregations at both the large and small mass limits in one dimension. To begin, our assumptions are E is constant in space and time, and all the organisms are in the same state i.e. $\bar{\rho} = \int \rho d\boldsymbol{n} \approx \rho \Delta n^N$ where N is the number of state dimensions and Δn represents a small area in state space. We also assume that the change in state is far slower than the movement of the organisms. Finally, we will label the support of ρ as Ω'. These assumptions allow is to rewrite (1) as,

$$\frac{1}{\Delta n^N}\frac{\partial \bar{\rho}}{\partial t} + \frac{1}{\Delta n^N}\nabla \cdot (\bar{\rho}\boldsymbol{v}_x) - \frac{1}{\Delta n^N}\nabla \cdot (D f_l(\boldsymbol{n}, E)\nabla \bar{\rho}) = 0,$$

with

$$\boldsymbol{v}_x = -\nabla(f_n(\boldsymbol{n}, E) Q * \bar{\rho}) - D f_l(\boldsymbol{n}, E)\gamma \nabla \bar{\rho}^2.$$

This can be rewritten as

$$\frac{\partial \bar{\rho}}{\partial t} + \nabla \cdot (\bar{\rho} \hat{\boldsymbol{v}}_x) = 0,$$

with

$$\hat{\boldsymbol{v}}_x = -\nabla \left[(f_n(\boldsymbol{n}, E) Q * \bar{\rho}) + D f_l(\boldsymbol{n}, E) \gamma \bar{\rho}^2 + D f_l(\boldsymbol{n}, E) \log(\bar{\rho}) \right].$$

This is a gradient flow [1] of the form

$$\frac{\partial \bar{\rho}}{\partial t} = \nabla \cdot \left(\bar{\rho} \nabla \left[\frac{\delta \mathcal{E}}{\delta \bar{\rho}} \right] \right),$$

where

$$\mathcal{E}[\bar{\rho}] = \int_{\Omega'} \frac{1}{2} \bar{\rho} [f_n(\boldsymbol{n}, E) Q * \bar{\rho}] + \frac{D f_l(\boldsymbol{n}, E) \gamma}{3} \bar{\rho}^3 + D f_l(\boldsymbol{n}, E) (\bar{\rho} \log(\bar{\rho}) - \bar{\rho}) \, dx, \tag{5}$$

with the minimisers satisfying

$$\frac{\delta \mathcal{E}}{\delta \rho} = (f_n(\boldsymbol{n}, E) Q * \bar{\rho}) + D f_l(\boldsymbol{n}, E) \gamma \bar{\rho}^2 + D f_l(\boldsymbol{n}, E) \log(\bar{\rho}) \ = \lambda.$$

We should note that either the variation in energy (given by (5)) will be zero (in regions with organisms) or the density $\bar{\rho}$ will be zero. In the next sections, we follow the work of [2, 3, 5] and with further simplifying assumptions we consider the maximum density and support of aggregations with small and large numbers of organisms (i.e. as $M \to 0$ and $M \to \infty$, respectively). We term these the small mass limit and large mass limit, respectively.

1.1.1 Large mass limit

We begin with (5) and then further assume the following: $\bar{\rho}(x)$ is approximately rectangular. Additionally, while the support of $\bar{\rho}$, Ω', is infinite due to the linear diffusion the bulk of the mass is contained as a series of aggregations, we will approximate the finite support of a single aggregation as Ω. Finally, for a single aggregation we assume that the support is far larger than the range of Q. We thus approximate $Q \approx V_Q \delta(x)$, where $\delta(x)$ is the Dirac delta function. We note that the $V_Q \delta(x)$ needs to preserve the volume of Q so $V_Q = \int Q d\boldsymbol{x}$. Then as $\bar{\rho}$ is rectangular

$$||\Omega|| = \frac{M}{\bar{\rho}},$$

where M is given by (4). Substituting into (5) we get

$$\mathcal{E}[\bar{\rho}] = M \left(\frac{V_Q}{2} f_n(\boldsymbol{n}, E) \bar{\rho} + \frac{D f_l(\boldsymbol{n}, E) \gamma}{3} \bar{\rho}^2 + D f_l(\boldsymbol{n}, E) (\log(\bar{\rho}) - 1) \right).$$

We can then find

$$\frac{dE}{d\bar{\rho}} = M \left(\frac{V_Q}{2} f_n(\boldsymbol{n}, E) + \frac{2 D f_l(\boldsymbol{n}, E) \gamma}{3} \bar{\rho} + \frac{D f_l(\boldsymbol{n}, E)}{\bar{\rho}} \right),$$

which has critical point at

$$\frac{V_Q}{2} f_n(\boldsymbol{n}, E) + \frac{2 D f_l(\boldsymbol{n}, E) \gamma}{3} \bar{\rho} + \frac{D f_l(\boldsymbol{n}, E)}{\bar{\rho}} = 0.$$

Thus

$$\bar{\rho} = \frac{-\frac{V_Q}{2} f_n(\boldsymbol{n}, E) \pm \sqrt{\left(\frac{V_Q}{2} f_n(\boldsymbol{n}, E) \right)^2 - \frac{8 (D f_l(\boldsymbol{n}, E))^2 \gamma}{3}}}{\frac{4}{3} D \gamma f_l(\boldsymbol{n}, E)},$$

which simplifies to

$$\bar{\rho} = -\frac{3V_Q}{8D\gamma}F \pm \sqrt{\left(\frac{3V_Q}{8D\gamma}\right)^2 F^2 - \frac{3}{2\gamma}},$$

where

$$F = \frac{f_n(\boldsymbol{n}, E)}{f_l(\boldsymbol{n}, E)}, \tag{6}$$

is the ratio of our non-local and local forces. We then take only the positive root, giving

$$||\bar{\rho}||_\infty = -\frac{3V_Q}{8D\gamma}F + \sqrt{\left(\frac{3V_Q}{8D\gamma}\right)^2 F^2 - \frac{3}{2\gamma}}, \tag{7}$$

with support

$$||\Omega|| = \frac{M}{-\frac{3V_Q}{8D\gamma}F + \sqrt{\left(\frac{3V_Q}{8D\gamma}\right)^2 F^2 - \frac{3}{2\gamma}}}. \tag{8}$$

There are a few things to note in these equations. Firstly for aggregations to exist, i.e. $||\bar{\rho}||_\infty > 0$, F must be less than 0 corresponding to an attractive social potential (or an attractive local movement; however, if both local and non-local components are attractive our original assumption of $\bar{\rho}$ being rectangular would not hold as the equation is singular). Next, from this we find that any change in state, $\boldsymbol{n}$, that increases the rate of local movement without a corresponding increase in non-local movement would decrease the maximum density of aggregations and thus increase the size of the support (and vice versa). Finally, any change in state that increases the rate of non-local movement compared to local movement would increase the maximum density of aggregations and thus decrease the size of the support.

We can also use (**??**) to estimate the parameter γ, given a maximum density of organisms, ρ_∞, we find,

$$\gamma = -\frac{6V_Q}{8D\rho_\infty}F - \frac{3}{2\rho_\infty^2}. \tag{9}$$

1.1.2 Small mass limit

Assuming that for a single aggregation we can approximate the social interaction potential using a Taylor expansion. In this section we use $Q(x) = e^{-\frac{|x|}{r}}$, with $e^{-\frac{|x|}{r}} \approx 1 - \frac{|x|}{r}$. Additionally, we ignore the effect of linear diffusion within Ω, giving (5) as

$$\mathcal{E}[\bar{\rho}] = \int_\Omega \frac{f_n(\boldsymbol{n}, E)}{2}\bar{\rho}[Q * \bar{\rho}] + \frac{D\gamma f_l(\boldsymbol{n}, E)}{3}\bar{\rho}^3\, dx. \tag{10}$$

Based on these assumptions we can find

$$\frac{\delta\mathcal{E}}{\delta\bar{\rho}} = \left(f_n(\boldsymbol{n}, E) - f_n(\boldsymbol{n}, E)\frac{|x|}{r}\right) * \bar{\rho} + D\gamma f_l(\boldsymbol{n}, E)\bar{\rho}^2 = \lambda,$$

which becomes

$$f_n(\boldsymbol{n}, E)M - \frac{f_n(\boldsymbol{n}, E)}{r}(|x| * \bar{\rho}) + D\gamma f_l(\boldsymbol{n}, E)\bar{\rho}^2 = \lambda.$$

We then exploit the property that $(|x|)_{xx} = 2\delta(x)$ and differentiate twice to obtain

$$-2\frac{f_n(\boldsymbol{n}, E)}{r}\bar{\rho} + D\gamma f_l(\boldsymbol{n}, E)(\bar{\rho}^2)_{xx} = 0.$$

Following [2] we place the maximum of $\bar{\rho}$ at the origin; this implies $\bar{\rho}_x(0) = 0$ and $\bar{\rho}(0) = ||\bar{\rho}||_\infty$. We then let,

$$p = \frac{\bar{\rho}}{||\bar{\rho}||_\infty}, \text{ and } \zeta = \frac{x}{\sqrt{||\bar{\rho}||_\infty}}, \tag{11}$$

giving,

$$(p^2)_{\zeta\zeta} - \frac{2f_n(\boldsymbol{n},E)}{Dr\gamma f_l(\boldsymbol{n},E)}p = 0,\ p(0) = 1,\ p_\zeta(0) = 0.$$

We then multiply through by $(p^2)_\zeta$ and integrate to obtain,

$$2p^2(p)_\zeta^2 - \frac{8f_n(\boldsymbol{n},E)}{3Dr\gamma f_l(\boldsymbol{n},E)}p^3 + c = 0.$$

Then applying the conditions at $\zeta = 0$ we find,

$$2p^2(p)_\zeta^2 - \frac{8f_n(\boldsymbol{n},E)}{3Dr\gamma f_l(\boldsymbol{n},E)}(p^3 - 1) = 0,$$

which can be simplified to,

$$(p)_\zeta = \sqrt{\frac{-2f_n(\boldsymbol{n},E)}{3Dr\gamma f_l(\boldsymbol{n},E)}\left(\frac{1}{p^2} - p\right)}.$$

Performing a separation of variables gives

$$d\zeta = \sqrt{\frac{3Dr\gamma f_l(\boldsymbol{n},E)}{-2f_n(\boldsymbol{n},E)}}\frac{p\,dp}{\sqrt{1-p^3}}. \tag{12}$$

We can then find the implicit solution,

$$\zeta = \sqrt{\frac{3Dr\gamma f_l(\boldsymbol{n},E)}{-2f_n(\boldsymbol{n},E)}}\int_p^1 \frac{p\,dp}{\sqrt{1-p^3}}.$$

As $p \to 0$, $\zeta \to \frac{||\Omega||}{2\sqrt{||\bar{\rho}||_\infty}}$, giving

$$\begin{aligned}
||\Omega|| &= 2\sqrt{||\bar{\rho}||_\infty}\sqrt{\frac{3Dr\gamma f_l(\boldsymbol{n},E)}{-2f_n(\boldsymbol{n},E))}}\int_0^1 \frac{p\,dp}{\sqrt{1-p^3}} \\
&= 2\sqrt{||\bar{\rho}||_\infty}\sqrt{\frac{3Dr\gamma f_l(\boldsymbol{n},E)}{-2f_n(\boldsymbol{n},E)}}\frac{1}{3}B\left(\frac{2}{3},\frac{1}{2}\right) \\
&= \sqrt{||\bar{\rho}||_\infty\frac{2Dr\gamma f_l(\boldsymbol{n},E)}{-3f_n(\boldsymbol{n},E)}}B\left(\frac{2}{3},\frac{1}{2}\right),
\end{aligned} \tag{13}$$

where B is the β-function (for definition see [7], page 207). Next using the mass constraint,

$$M = 2\int_0^{\frac{||\Omega||}{2}} \bar{\rho}(x)\,dx = 2\int_{-\frac{||\Omega||}{2}}^0 \bar{\rho}(x)\,dx,$$

and substituting (11) we obtain

$$M = 2||\bar{\rho}||_\infty^{\frac{3}{2}}\int_{-\frac{||\Omega||}{2\sqrt{||\bar{\rho}||_\infty}}}^0 p(\zeta)\,d\zeta,$$

which using (12) becomes,

$$\begin{aligned}
M &= ||\bar{\rho}||_\infty^{\frac{3}{2}}\sqrt{\frac{6Dr\gamma f_l(\boldsymbol{n},E)}{-f_n(\boldsymbol{n},E)}}\int_0^1 \frac{p^2\,dp}{\sqrt{1-p^3}} \\
&= ||\bar{\rho}||_\infty^{\frac{3}{2}}\sqrt{\frac{8Dr\gamma f_l(\boldsymbol{n},E)}{-3f_n(\boldsymbol{n},E)}}.
\end{aligned} \tag{14}$$

Then using (13) and (14) we can find $||\Omega||$ and $||\bar{\rho}||_\infty$ in terms of M and F ((4) and (6), respectively) giving

$$||\bar{\rho}||_\infty = \sqrt[3]{\frac{-3M^2F}{8Dr\gamma}}, \tag{15}$$

and

$$||\Omega|| = B\left(\frac{2}{3}, \frac{1}{2}\right)\sqrt[3]{\frac{MDr\gamma}{-3F}}, \tag{16}$$

which gives a similar relationship as the large mass limit.

1.1.3 Comparison of mass limits and simulations

We can check the accuracy of our estimates by comparing them to simulation results. We begin by defining two dimensional state space, (n_g, n_h), with the first dimension only affecting the non-local component of force and the second dimension affecting the local component. For example, these could be considered in locusts to be dimensions of gregarisation and hunger, respectively. We first let,

$$f_n(\boldsymbol{n}) = 2\left(0.5 - \frac{1}{1 + e^{-15(n_1 - 0.5)}}\right),$$

and

$$f_l(\boldsymbol{n}, E) = (1.5 - n_2),$$

in addition we let,

$$Q(x) = e^{-|x|},\ D = 0.01,\ \text{and}\ \gamma = 60.$$

For each simulation we place a mass of organisms, M, in every combination of the states $n_g = 0.75$, 0.85, 0.95 and $n_h = 0.15$, 0.55, 0.95 in the center of the domain (we vary the domain size according to mass so there is no boundary interaction) and run it to a pseudo steady state, $t = 1000$.

The results for $||\bar{\rho}||_\infty$ can be seen in Figure 1 and the results for $||\Omega||$ can be seen in Figure 2. In the plots, the dotted lines represent the minimum estimates between the small and large mass limits, and the solid lines represent simulated results. As $||\Omega||$ is theoretically infinite due to the linear diffusion, for the simulated $||\Omega||$ we select the region for which 98% of the mass, M, is contained. We can see that as M increases the simulated limits approach those given by the theoretical estimates, however there is some error likely due to error in the simulations and our method of approximating $||\Omega||$. In addition, the small mass estimates are considerably less accurate than the large mass limits (due to ignoring linear diffusion), however, the estimates display qualitatively similar behaviour.

1.2 Linear stability analysis of homogeneous steady states

In order to gain insights into the conditions under which aggregations can form, we investigate the stability of spatially-homogeneous steady states. In this analysis we perturb the homogeneous steady state by adding a small amount of noise. We then find under what conditions the small perturbations grow and are likely to lead to aggregations. We again assume that E is constant in space and time.

1.2.1 A single state

We begin by considering all organisms in a single state, $\boldsymbol{n}_f$, i.e. $\bar{\rho} = \int \rho d\boldsymbol{n} \approx \rho \Delta n^m$ where m is the number of state dimensions. We also assume that the change in state is of the order ϵ^2 where $\epsilon \ll 1$. These assumptions give,

$$\frac{1}{\Delta n^m}\frac{\partial \bar{\rho}}{\partial t} + \frac{1}{\Delta n^m}\nabla \cdot (\bar{\rho}\boldsymbol{v}_x) + \frac{\epsilon^2}{\Delta n^m}\nabla_n \cdot (\bar{\rho}\boldsymbol{v}_n) - \frac{1}{\Delta n^m}\nabla \cdot (Df_l(\boldsymbol{n}, E)\nabla\bar{\rho}) = 0,$$

with

$$\boldsymbol{v}_x = -\nabla(f_n(\boldsymbol{n}, E)Q * \bar{\rho}) - Df_l(\boldsymbol{n}, E)\gamma\nabla\bar{\rho}^2,$$

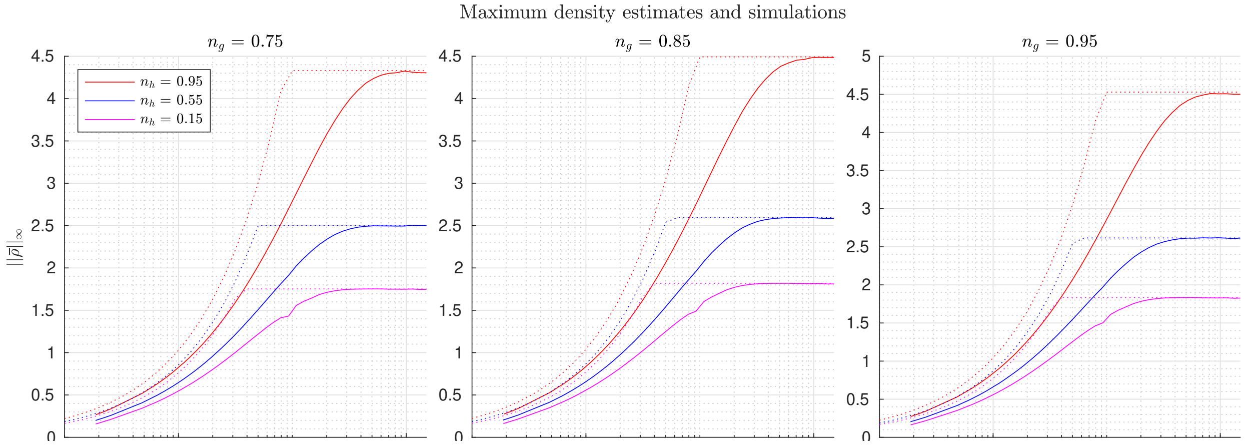

Figure 1: **Small and large mass density estimates and simulations.** Estimates for the small and large mass density limits in different states are plotted in dotted lines, with the corresponding simulation results plotted in solid lines.

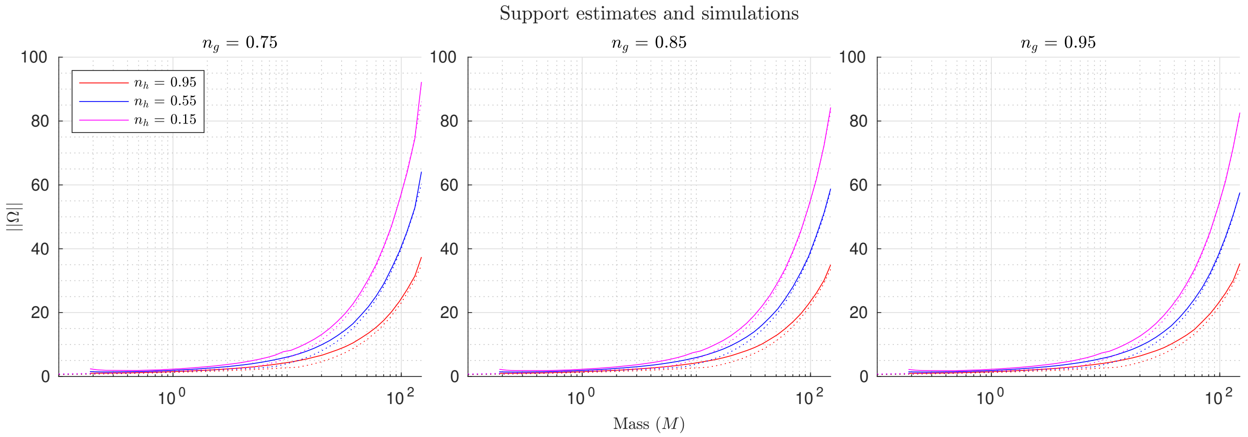

Figure 2: **Small and large mass max density and support, estimates and simulations.** Estimates and simulations for the maximum density (left) ans size of the support (right). Estimates for different states are plotted in dotted lines, with the corresponding simulation results plotted in solid lines. As $||\Omega||$ is theoretically infinite due to the linear diffusion, for the simulated $||\Omega||$ we select the region for which 98% of the mass, M, is contained.

which then becomes

$$\frac{\partial \bar{\rho}}{\partial t} + \nabla \cdot (\bar{\rho} \boldsymbol{v}_x) + \epsilon^2 \nabla_n \cdot (\bar{\rho} \boldsymbol{v}_n) - \nabla \cdot (D f_l(\boldsymbol{n}_f, E) \nabla \bar{\rho}) = 0, \tag{17}$$

with

$$\boldsymbol{v}_x = -\nabla (f_n(\boldsymbol{n}_f) Q * \bar{\rho}) - D f_l(\boldsymbol{n}_f, E) \gamma \nabla \bar{\rho}^2.$$

We then perturb around a homogeneous steady state, by letting $\bar{\rho} = \rho_c + \epsilon \tilde{\rho}$ where ρ_c is the homogeneous steady state and $\epsilon \tilde{\rho}$ is a small perturbation ($\epsilon \ll 1$). Substituting this into (17) and removing terms of $\mathcal{O}(\epsilon^2)$, we find

$$\epsilon \frac{\partial \tilde{\rho}}{\partial t} + \nabla \cdot (\epsilon \rho_c \boldsymbol{v}_x) - \nabla \cdot (D f_l(\boldsymbol{n}_f, E) \nabla (\epsilon \tilde{\rho})) = 0,$$

with

$$\boldsymbol{v}_x(\boldsymbol{n}) = -f_n(\boldsymbol{n}, E)(Q * \nabla \tilde{\rho}) - D \gamma f_l(\boldsymbol{n}_f, E) \nabla (\rho_c \tilde{\rho}).$$

Next we take Fourier transforms in space and Laplace transforms in time of $\tilde{\rho}$, i.e. $\tilde{\rho} \propto e^{-ikx+\lambda t}$ to obtain

$$\epsilon\lambda\tilde{\rho} + \left[k^2 f_n(\boldsymbol{n}_f)(\hat{Q}\tilde{\rho}) + Dk^2\gamma f_l(\boldsymbol{n}_f, E)\rho_c\tilde{\rho}\right]\epsilon\rho_c = Dk^2\left[f_l(\boldsymbol{n}_f, E)\epsilon\tilde{\rho}\right],$$

where $\hat{Q}$ is the Fourier transform of Q. Then, dividing through by $\epsilon\tilde{\rho}$ we get

$$\lambda = -\left[k^2 f_n(\boldsymbol{n}_f)(\hat{Q}) + Dk^2\gamma f_l(\boldsymbol{n}_f, E)\rho_c\right]\rho_c - Dk^2\left[f_l(\boldsymbol{n}_f, E)\right].$$

If $\lambda > 0$ then small perturbations will grow in time, we can then find the condition for instability in terms of our two state based force, as

$$-f_n(\boldsymbol{n}_f)\hat{Q} > \left(D\gamma\rho_c + \frac{D}{\rho_c}\right) f_l(\boldsymbol{n}_f, E), \tag{18}$$

or alternatively

$$\frac{-f_n(\boldsymbol{n}_f)\hat{Q}}{\left(D\gamma\rho_c + \frac{D}{\rho_c}\right) f_l(\boldsymbol{n}_f, E)} > 1. \tag{19}$$

From this we can see that if $f_n(\boldsymbol{n}_f) > 0$, corresponding to a repulsive non-local term, then aggregations will not form and if $f_n(\boldsymbol{n}_f) < 0$ then non-local forces simply need to be greater than local forces for aggregations to form.

1.2.2 Two states

We can also consider two distinct sub-populations in states $\boldsymbol{n}_S$ and $\boldsymbol{n}_G$ with $f_n(\boldsymbol{n}_G, E) < f_n(\boldsymbol{n}_S, E)$ (i.e. $f_n(\boldsymbol{n}_G, E)$ is less repulsive than $f_n(\boldsymbol{n}_S, E)$, or even attractive). The fraction of the population in state $\boldsymbol{n}_G$ is given by ϕ_g. This allows us to rephrase (1) as a system of PDEs given by,

$$\phi_g\frac{\partial\bar{\rho}}{\partial t} + \phi_g\nabla\cdot(\boldsymbol{v}_x(\boldsymbol{n}_G)\bar{\rho}) + \epsilon^2\phi_g\nabla_n\cdot(\bar{\rho}\boldsymbol{v}_n) = \phi_g D\nabla\cdot\left[f_l(\boldsymbol{n}_G, E)\nabla\bar{\rho}\right], \tag{20}$$

$$(1-\phi_g)\frac{\partial\bar{\rho}}{\partial t} + (1-\phi_g)\nabla\cdot(\boldsymbol{v}_x(\boldsymbol{n}_S)\bar{\rho})) + \epsilon^2(1-\phi_g)\nabla_n\cdot(\bar{\rho}\boldsymbol{v}_n) = (1-\phi_g)D\nabla\cdot\left[f_l(\boldsymbol{n}_S, E)\nabla\bar{\rho}\right], \tag{21}$$

with

$$\boldsymbol{v}_x(\boldsymbol{n}) = -\nabla(f_n(\boldsymbol{n}, E)Q * \bar{\rho}) - D\gamma f_l(\boldsymbol{n}, E)\nabla\left(\bar{\rho}^2\right).$$

As in the single state case, we then perturb around a homogeneous steady state, by letting $\bar{\rho} = \rho_c + \epsilon\tilde{\rho}$ where ρ_c is the homogeneous steady state and $\epsilon\tilde{\rho}$ is a small perturbation ($\epsilon \ll 1$). Substituting this into (20) and (21), removing terms of $\mathcal{O}(\epsilon^2)$, then adding (20) and (21) together we find

$$\begin{aligned}\epsilon\frac{\partial\tilde{\rho}}{\partial t} &+ \nabla\cdot\left[\left(\phi_g\boldsymbol{v}_x(\boldsymbol{n}_G) + (1-\phi_g)\boldsymbol{v}_x(\boldsymbol{n}_S)\right)(\rho_c + \epsilon\tilde{\rho})\right]\\ &= D\nabla\cdot\left[\left(\phi_g f_l(\boldsymbol{n}_G, E) + (1-\phi_g)f_l(\boldsymbol{n}_S, E)\right)\epsilon\nabla\tilde{\rho}\right],\end{aligned}$$

with

$$\boldsymbol{v}_x(\boldsymbol{n}) = -\epsilon f_n(\boldsymbol{n}, E)(Q * \nabla\tilde{\rho}) - D\gamma f_l(\boldsymbol{n}, E)\nabla\left(\epsilon\rho_c\tilde{\rho}\right).$$

This can be simplified further into (by removing the next bunch of $\mathcal{O}(\epsilon^2)$),

$$\begin{aligned}\epsilon\frac{\partial\tilde{\rho}}{\partial t} &+ \nabla\cdot\left[\left(\phi_g\boldsymbol{v}_x(\boldsymbol{n}_G) + (1-\phi_g)\boldsymbol{v}_x(\boldsymbol{n}_S)\right)\epsilon\rho_c\right]\\ &= D\nabla\cdot\left[\left(\phi_g f_l(\boldsymbol{n}_G, E) + (1-\phi_g)f_l(\boldsymbol{n}_S, E)\right)\epsilon\nabla\tilde{\rho}\right],\end{aligned}$$

with

$$\boldsymbol{v}_x(\boldsymbol{n}) = -f_n(\boldsymbol{n}, E)(Q * \nabla\tilde{\rho}) - D\gamma f_l(\boldsymbol{n}, E)\nabla\left(\rho_c\tilde{\rho}\right).$$

Next we take Fourier transforms in space and Laplace transforms in time of $\tilde{\rho}$, i.e. $\tilde{\rho} \propto e^{-ikx+\lambda t}$ to obtain

$$\begin{aligned}
&\epsilon\lambda\tilde{\rho} + \phi_g \left[k^2 f_n(\boldsymbol{n}_G, E)(\hat{Q}\tilde{\rho}) - Dk^2\gamma f_l(\boldsymbol{n}_G, E)\rho_c\tilde{\rho}\right]\epsilon\rho_c \\
&\quad + (1-\phi_g)\left[k^2 f_n(\boldsymbol{n}_S, E)(\hat{Q}\tilde{\rho}) - Dk^2\gamma f_l(\boldsymbol{n}_S, E)\rho_c\tilde{\rho}\right]\epsilon\rho_c \\
&\quad = -Dk^2\left[(\phi_g f_l(\boldsymbol{n}_G, E) + (1-\phi_g)f_l(\boldsymbol{n}_S, E))\,\epsilon\tilde{\rho}\right],
\end{aligned}$$

where $\hat{Q}$ is the Fourier transform of Q. Then, dividing through by $\epsilon\tilde{\rho}$ we get

$$\begin{aligned}
\lambda = &-\phi_g\left[k^2 f_n(\boldsymbol{n}_G, E)\hat{Q} - Dk^2\gamma f_l(\boldsymbol{n}_G, E)\rho_c\right]\rho_c \\
&-(1-\phi_g)\left[k^2 f_n(\boldsymbol{n}_S, E)\hat{Q} - Dk^2\gamma f_l(\boldsymbol{n}_S, E)\rho_c\right]\rho_c \\
&- Dk^2\left[\phi_g f_l(\boldsymbol{n}_G, E) + (1-\phi_g)f_l(\boldsymbol{n}_S, E)\right].
\end{aligned}$$

If $\lambda > 0$ then small perturbations will grow in time, we can then find the condition for instability in terms of ϕ_g as

$$\phi_g > \phi_g^* = \frac{f_n(\boldsymbol{n}_S, E)\hat{Q} + D\gamma f_l(\boldsymbol{n}_S, E)\rho_c + \frac{Df_l(\boldsymbol{n}_S,E)}{\rho_c}}{\hat{Q}A_n + (D\gamma\rho_c + \frac{D}{\rho_c})A_l)}, \tag{22}$$

where

$$A_n(f_n(\boldsymbol{n}_S, E) - f_n(\boldsymbol{n}_G, E), \text{ and } A_l = (f_l(\boldsymbol{n}_S, E) - f_l(\boldsymbol{n}_G, E).$$

From this we can see that: in environmental conditions that reduce dispersal the gregarious fraction required for aggregation formation decreases. The numerator is only in terms of $\boldsymbol{n}_S$, thus states that increase the local and non-local terms will increase the gregarious mass fraction required for aggregation formation. The denominator depends on the difference between the states: Locally, if the gregarious state has less local movement than the solitarious state this decreases the gregarious mass fraction required for aggregation formation and vice-versa. In addition, if the two local forces are equal, $f_l(\boldsymbol{n}_G) = f_l(\boldsymbol{n}_S)$, this reduces to the stability condition found in our previous two population model [4], and if the local forces are 0 it reduces to that of Topaz et al. [6]. Non-locally, decreasing the non-local force of both the gregarious and solitarious states decreases the gregarious mass fraction required for aggregation formation and vice-versa (as $f_n < 0$ is attraction and $f_n(\boldsymbol{n}_G, E) < f_n(\boldsymbol{n}_S, E)$). Finally, as $\bar{\rho}$ increases the gregarious fraction required for aggregation formation increases suggesting an upper organism density in order to transition away from the homogeneous steady state.

For our specific function $Q = e^{-\frac{|x|}{r}}$, we begin by taking the one dimensional Fourier transforms of Q using the following definition,

$$\hat{f}(\hat{k}) = \int_{\mathbb{R}^n} f(\boldsymbol{x})e^{-i\hat{k}\cdot\boldsymbol{x}}\,d\boldsymbol{x},$$

to get

$$\hat{Q} = \frac{2r}{1+r^2\hat{k}^2}. \tag{23}$$

We must now consider the conditions on $\hat{k}$ that produce the lowest ϕ_g^* in (22), corresponding to the first wave number to begin producing aggregations. To start we want to minimise

$$\phi_g^*(\hat{k}) = \frac{f_n(\boldsymbol{n}_S, E)\hat{Q} + D\gamma f_l(\boldsymbol{n}_S, E)\rho_c + \frac{Df_l(\boldsymbol{n}_S,E)}{\rho_c}}{\hat{Q}A_n + (D\gamma\rho_c + \frac{D}{\rho_c})A_l)}. \tag{24}$$

We do this by finding $\frac{d\phi_g^*(\hat{k})}{d\hat{k}} = 0$ which simplifies to

$$\frac{d\hat{Q}}{d\hat{k}}(f_l(\boldsymbol{n}_S, E)f_n(\boldsymbol{n}_G, E) - f_l(\boldsymbol{n}_G, E)f_n(\boldsymbol{n}_S, E)) = 0, \tag{25}$$

$$\frac{-4r^3k(f_l(\boldsymbol{n}_S, E)f_n(\boldsymbol{n}_G, E) - f_l(\boldsymbol{n}_G, E)f_n(\boldsymbol{n}_S, E))}{(1+r^2k^2)^2} = 0. \tag{26}$$

Then, provided

$$\frac{f_n(\boldsymbol{n}_G,E)}{f_l(\boldsymbol{n}_G,E)} < \frac{f_n(\boldsymbol{n}_S,E)}{f_l(\boldsymbol{n}_S,E)},$$

$\phi_g^*(\hat{k})$ has a minimum value at $\hat{k}=0$. If

$$\frac{f_n(\boldsymbol{n}_G,E)}{f_l(\boldsymbol{n}_G,E)} = \frac{f_n(\boldsymbol{n}_S,E)}{f_l(\boldsymbol{n}_S,E)},$$

$\phi_g^*(\hat{k})$ is constant in $\hat{k}$ and we cannot say which wave number would lead to instability. Finally, if

$$\frac{f_n(\boldsymbol{n}_G,E)}{f_l(\boldsymbol{n}_G,E)} > \frac{f_n(\boldsymbol{n}_S,E)}{f_l(\boldsymbol{n}_S,E)},$$

$\phi_g^*(\hat{k})$ has a maximum value at $\hat{k}=0$ and as $\hat{Q}(\hat{k})$ is monotonic in $\hat{k}$ we need to look at the limit as $\hat{k}\to\infty$. We will consider the inequalities in turn.

1.2.3 $\hat{k}=0$

To start, let $\hat{k}=0$ and substitute into (22), which gives,

$$\phi_g > \phi_g^* = \frac{2rf_n(\boldsymbol{n}_S,E)+Df_l(\boldsymbol{n}_S,E)\rho_c+\frac{Df_l(\boldsymbol{n}_S,E)}{\rho_c}}{2rA_n+(D\gamma\rho_c+\frac{D}{\rho_c})A_l)}, \tag{27}$$

where

$$A+n=(f_n(\boldsymbol{n}_S,E)-f_n(\boldsymbol{n}_G,E), \text{ and } A_l=(f_l(\boldsymbol{n}_S,E)-f_l(\boldsymbol{n}_G,E).$$

From this we can find the maximum homogeneous density, ρ_c, that aggregations can still form. So taking (**??**) and substituting $\phi_g=1$ gives,

$$1=\frac{2rf_n(\boldsymbol{n}_S,E)+Df_l(\boldsymbol{n}_S,E)\rho_c+\frac{Df_l(\boldsymbol{n}_S,E)}{\rho_c}}{2rA+(D\gamma\rho_c+\frac{D}{\rho_c})B)}.$$

This gives

$$Df_l(\boldsymbol{n}_G,E)\rho_c^2-2rf_n(\boldsymbol{n}_G,E)\rho_c+Df_l(\boldsymbol{n}_G,E)=0,$$

and this has solutions

$$\rho_c=-\frac{r}{D\gamma}F+\sqrt{\left(\frac{r}{D\gamma}\right)^2F^2-\frac{1}{\gamma}},$$

where F is given by (**??**) for the state $\boldsymbol{n}_G$. Rather neatly, if $r=\frac{1}{2}$, corresponding to $\int_{\Omega_x}Q(x)dx=1$, this becomes

$$\rho_c\approx\frac{2}{3}||\rho||_\infty,$$

where $||\rho||_\infty$ is given by (**??**), this is similar to the relationship previously derived in [4]. Finally, we note that the upper limit of aggregation formation only depends on the forces of organisms in the gregarious state.

1.2.4 $\hat{k}\to\infty$

To start, let $\hat{k}\to\infty$ and substitute into (22), which gives,

$$\phi_g > \phi_g^* = \frac{f_l(\boldsymbol{n}_S,E)}{f_l(\boldsymbol{n}_S,E)-f_l(\boldsymbol{n}_G,E)}, \tag{28}$$

which only depends on the local state based forces of the two populations. Then as

$$\frac{f_n(\boldsymbol{n}_G, E)}{f_l(\boldsymbol{n}_G, E)} > \frac{f_n(\boldsymbol{n}_S, E)}{f_l(\boldsymbol{n}_S, E)},$$

and $f_n(\boldsymbol{n}_G, E) < f_n(\boldsymbol{n}_S, E)$ if the signs of $f_n(\boldsymbol{n}_G, E)$ and $f_n(\boldsymbol{n}_S, E)$ are the same then this implies $f_l(\boldsymbol{n}_G, E)$ must be less than $f_l(\boldsymbol{n}_S, E)$ further implying that $\phi_g^* > 1$. Therefore aggregations will not form within this state regime.

References

[1] Luigi Ambrosio, Nicola Gigli, and Giuseppe Savare. ***Gradient Flows: In Metric Spaces and in the Space of Probability Measures***. en. 2nd ed. Lectures in Mathematics. ETH Zürich. Birkhäuser Basel, 2008. ISBN: 978-3-7643-8721-1. URL: `https://www.springer.com/gp/book/9783764387211`.

[2] A. J. Bernoff and C. M. Topaz. "Biological Aggregation Driven by Social and Environmental Factors: A Nonlocal Model and Its Degenerate Cahn–Hilliard Approximation". In: ***SIAM Journal on Applied Dynamical Systems*** 15.3 (Jan. 2016), pp. 1528–1562. DOI: `10.1137/15M1031151`.

[3] M. Burger, R. Fetecau, and Y. Huang. "Stationary States and Asymptotic Behavior of Aggregation Models with Nonlinear Local Repulsion". In: ***SIAM Journal on Applied Dynamical Systems*** 13.1 (Jan. 2014), pp. 397–424. DOI: `10.1137/130923786`.

[4] Fillipe Georgiou et al. "Modelling locust foraging: How and why food affects group formation". In: ***PLOS Computational Biology*** 17.7 (July 2021), e1008353. ISSN: 1553-7358. DOI: `10.1371/journal.pcbi.1008353`.

[5] C. M. Topaz, A. L. Bertozzi, and M. A. Lewis. "A Nonlocal Continuum Model for Biological Aggregation". In: ***Bulletin of Mathematical Biology*** 68.7 (July 2006), p. 1601. ISSN: 1522-9602. DOI: `10.1007/s11538-006-9088-6`.

[6] C. M. Topaz et al. "Locust Dynamics: Behavioral Phase Change and Swarming". In: ***PLOS Computational Biology*** 8.8 (Aug. 2012), e1002642. ISSN: 1553-7358. DOI: `10.1371/journal.pcbi.1002642`.

[7] Eric W. Weisstein. ***CRC Concise Encyclopedia of Mathematics***. CRC Press, Dec. 2002. ISBN: 978-1-4200-3522-3.
