# Supplementary material for: Including population and environmental dynamic heterogeneities in continuum models of collective behaviour with applications to locust foraging and group structure
Source: PLoS Comput Biol. 2025 Apr 15;21(4):e1011469. doi: 10.1371/journal.pcbi.1011469 (PMC11999712; doi:10.1371/journal.pcbi.1011469)
Supplement: S3 Appendix — The full detailed derivation of the numerical scheme used for simulating the numerical results as well as some extra numerical results. (PDF) [file pcbi.1011469.s003.pdf]

S3 Appendix: Numerical Scheme

Fillipe Georgiou[1], Camille Buhl[2], J.E.F. Green[3],
Bishnu Lamichhane[4] and Ngamta Thamwattana[4]
[1] Institute for Mathematical Innovation, University of Bath,
Bath, United Kingdom.
[2] School of Agriculture, Food and Wine, University of Adelaide,
Adelaide, Australia.
[3] School of Computer & Mathematical Sciences, University of Adelaide,
Adelaide, Australia.
[4] School of Information and Physical Sciences, University of Newcastle,
Callaghan, Australia.

February 24, 2025

For this numerical scheme our full equation is given by:

$$\frac{\partial \rho}{\partial t} + \nabla \cdot (\boldsymbol{v}_x \rho) + \nabla_{\boldsymbol{n}} \cdot (\boldsymbol{v}_n \rho) = D \nabla \cdot \left[f_l(\boldsymbol{n}, E) \nabla \rho \right], \tag{1}$$

with

$$\boldsymbol{v}_x = -f_n(\boldsymbol{n}, E) \nabla (Q * \bar{\rho}) - D \left[\nabla f_l(\boldsymbol{n}, E) + \gamma f_l(\boldsymbol{n}, E) \nabla \left(\tau(\bar{\rho}) \right) \right], \tag{2}$$

and

$$\tau(\bar{\rho}) = \bar{\rho}^2. \tag{3}$$

1 Numerical Scheme

We now derive the numerical scheme for (1) in one spatial dimension using a finite volume method (FVM). In this section we use index notation inspired by Karlsen and Risebro [4], and Bürger et al. [1]. In this notation the arbitrary cell i is given by the tuple of vectors $i = (i_{\boldsymbol{x}}, i_{\boldsymbol{n}})$ with a spatial step indexed by $i \pm e_x$ and a state step indexed by $i \pm e_n$. This gives cell boundaries as $i \pm \frac{1}{2} e_x$ for space and $i \pm \frac{1}{2} e_n$ for state. We will also define

$$\varrho_i = \sum_{i_n} \rho_i.$$

For the numerical scheme the terms are described in Table 1.

Beginning with the local part of the velocity term (denoted $\mathscr{L}$)

$$\mathscr{L} = -D \left(\frac{\partial f_l(\boldsymbol{n}, E)}{\partial x} + f_l(\boldsymbol{n}, E) \gamma \frac{\partial \bar{\rho}^2}{\partial x} \right),$$

we approximate both derivatives using central differencing schemes, giving

$$\mathscr{L}_i \approx -D \left(\frac{f_l(n_i, E_{i+e_x}) - f_l(n_i, E_{i-e_x})}{2\Delta x} + \gamma f_l(n_i, E_i) \frac{\varrho^2_{i+e_x} - \varrho^2_{i-e_x}}{2\Delta x} \right),$$

Symbol	Definition
Δx	spatial size of cells
Δn	state size of cells
$\mathbf{x}$	vector representing the discretised spatial grid
$\mathbf{n}$	vector representing the discretised state grid
x_i	spatial value of the midpoint of a grid cell
n_i	state value of the midpoint of a grid cell
ρ_i	Approximate function values of ρ
ϱ_i	Approximate function values of $\bar{\rho}$
E_i	Approximate function values of E
$\boldsymbol{\varrho}$	vectors representing the discretised function ϱ
$\mathscr{L}_i$	Approximate value of the local component of the $\boldsymbol{v}_x$
$\mathscr{N}_i$	Approximate value of the non-local component of $\boldsymbol{v}_x$
$\mathscr{A}_i$	Approximate value of the advective component of the equation
$\mathscr{D}_i$	Approximate value of the diffusive component of the equation
$\mathscr{V}_i$	Approximate value of $\boldsymbol{v}_n$
$\mathscr{S}_i$	Approximate value of the change in state

Table 1: Definitions of symbols used in numerical scheme at arbitrary cell $i = (i_{\boldsymbol{x}}, i_{\boldsymbol{n}})$.

at an arbitrary cell i. Then, for the non-local component of the velocity term (denoted $\mathscr{N}$),

$$\mathscr{N} = -\nabla(Q * \rho),$$

we begin by exploiting the convolution theorem, which states that under suitable conditions the Fourier transform of a convolution of two functions is equal to the point-wise product of their individual Fourier transforms, i.e.,

$$\mathscr{F}\{f * g\} = \mathscr{F}\{f\} \cdot \mathscr{F}\{g\},$$

where $\mathscr{F}$ represents the Fourier transform (we also denote the inverse Fourier transform as $\mathscr{F}^{-1}$). Additionally, we use the following property of convolutions

$$\frac{\partial}{\partial x}(f * g) = \left(\left(\frac{\partial}{\partial x} f\right) * g\right) = \left(f * \left(\frac{\partial}{\partial x} g\right)\right),$$

to turn the convolution component of the advection term into

$$\mathscr{N} = f_n(\boldsymbol{n}, E)\mathscr{F}^{-1}\left\{\mathscr{F}\left\{-\frac{\partial}{\partial x} Q\right\} \cdot \mathscr{F}\left\{\bar{\rho}\right\}\right\}.$$

We can then approximate the convolution as

$$\mathscr{N} \approx \text{real}\left\{\text{ifft}\left\{\text{fft}\left\{-\frac{\partial}{\partial x} Q(\mathbf{x})\right\} \cdot \text{fft}\left\{\varrho\right\}\right\}\right\}, \tag{4}$$

where fft and ifft represent the fast Fourier transform and inverse fast Fourier transform respectively. We take only the real component of the ifft as any imaginary value will simply be due to error. By combining the local and non-local components and letting

$$F_i = (\mathscr{L}_i + f_n(n_i, E_i)\mathscr{N}_i)\rho_i,$$

we can approximate the wavespeed at a cell boundary, $i - \frac{1}{2}e_x$, as

$$\hat{S}_{i-\frac{1}{2}e_x} = (F_i - F_{i-e_x})\text{sign}(\rho_i - \rho_{i-e_x}),$$

and

$$\hat{W}_{i-\frac{1}{2}e_x} = \text{sign}(\rho_i - \rho_{i-e_x}),$$

where

$$\text{sign}(x) = \begin{cases} -1 & x < 0, \\ 0 & x = 0, \\ 1 & x > 0. \end{cases}$$

Giving the upwinding scheme for the advection component of movement as

$$\mathscr{A}_i = \frac{1}{\Delta x}\left(\max\{\hat{S}_{i-\frac{1}{2}e_x}, 0\}\hat{W}_{i-\frac{1}{2}e_x} + \min\{\hat{S}_{i+\frac{1}{2}e_x}, 0\}\hat{W}_{i+\frac{1}{2}e_x}\right).$$

Next, for the diffusion term, $\mathscr{D}$

$$\mathscr{D} = D\frac{\partial}{\partial x}\left[f_l(\boldsymbol{n}, E)\frac{\partial \rho}{\partial x}\right].$$

We can approximate this using FVM as

$$\mathscr{D}_i \approx \frac{D}{\Delta x^2}\left(f_l\left(n_i, \frac{E_{i-e_x} + E_i}{2}\right)(\rho_i - \rho_{i-e_x}) - f_l\left(n_i, \frac{E_{i+e_x} + E_i}{2}\right)(\rho_{i+e_x} - \rho_i)\right).$$

For the state component

$$\mathscr{S} = \nabla_{\boldsymbol{n}} \cdot (\boldsymbol{v}_n \rho),$$

we begin by directly calculating the wave speed at the cell boundaries

$$\hat{W}_{i-\frac{1}{2}e_n} = \mathscr{V}_{i-\frac{1}{2}e_n}.$$

We then find the flux through the cell boundary due to state velocity using the upwinding scheme

$$\hat{F}_{i-\frac{1}{2}e_n} = \rho_i \min(\hat{W}_{i-\frac{1}{2}e_n}, 0) + \rho_{i-e_n} \max(\hat{W}_{i-\frac{1}{2}e_n}, 0),$$

giving

$$\mathscr{S}_i = \frac{1}{\Delta n}(\hat{F}_{i+\frac{1}{2}e_n} - \hat{F}_{i-\frac{1}{2}e_n}).$$

Combining all the terms we obtain,

$$\rho_i^{t+\Delta t} = \rho_i^t - \Delta t(\mathscr{A}_i + \mathscr{D}_i + \mathscr{S}_i).$$

For the CPU case we use an adaptive Dormand-Prince method [2] for the time component and for the GPU case we use an adaptive scheme RK4 based on the work of Horsea and Shampine [3]. Finally, all code is available in the following git repository.

A brief summary of the simulation resolutions used is: All simulations used 20 grid cells for each dimension in state. For the mass experiments 1024 grid cells were used in space (due to the larger domain to avoid boundary interactions). Next, for the food footprint simulations with one state dimension, we used 512 grid cells in space. Finally, for two state dimensions 256 grid cells were used in space for most of the parameter range, for the range of ω from 5% to 15% we used 512 grid cells in space to minimise any error caused by the steep gradients at the edge of the food distribution.

2 Extra results

In the interest of completeness we have included a copy of Figure 3 from the main text with the no food results. This can be seen in Figure 1.

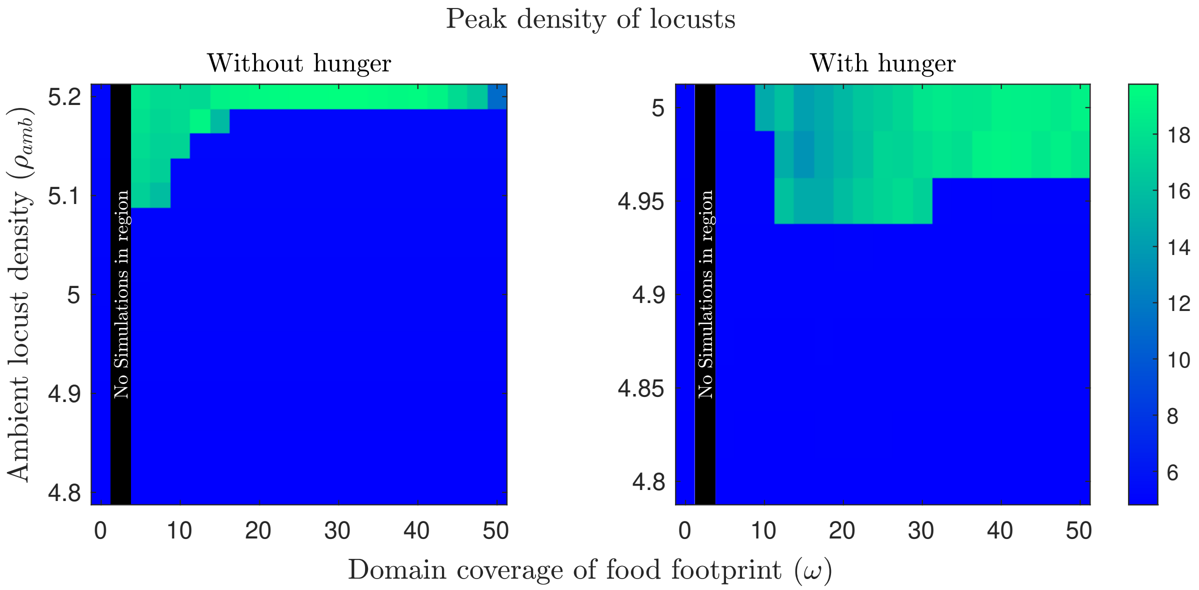

Figure 1: **Maximum locust density with varying food footprint sizes and initial ambient locust densities.** For the simulations, $x \in [0, 3]$ with periodic boundary conditions and $t \in [0, 10]$. Ambient locust density ranges from $\rho_{\text{amb}} = 4.8$ to $\rho_{\text{amb}} = 5.2$ in the case where hunger is absent and from $\rho_{\text{amb}} = 4.8$ to $\rho_{\text{amb}} = 5$ where hunger is present. Food footprint ranges from $\omega = 5\%$ to $\omega = 50\%$, including the simulations with $\omega = 0\%$. The plots show the maximum locust density at $t = 10$ for the varying food footprint sizes and ambient locust densities with a food mass of 1.

References

[1] R. Bürger et al. "Implicit-explicit methods for a class of nonlinear nonlocal gradient flow equations modelling collective behaviour". In: ***Applied Numerical Mathematics*** 144 (Oct. 2019), pp. 234–252. ISSN: 0168-9274. DOI: 10.1016/j.apnum.2019.04.018.

[2] J. R. Dormand and P. J. Prince. "A family of embedded Runge-Kutta formulae". In: ***Journal of Computational and Applied Mathematics*** 6.1 (Mar. 1980), pp. 19–26. ISSN: 0377-0427. DOI: 10.1016/0771-050X(80)90013-3.

[3] M.E. Hosea and L.F. Shampine. "Estimating the error of the classic Runge–Kutta Formula". In: ***Applied Mathematics and Computation*** 66.2–3 (Dec. 1994), pp. 217–226. ISSN: 0096-3003. DOI: 10.1016/0096-3003(94)90117-1.

[4] K.H. Karlsen and N.H. Risebro. "Convergence of finite difference schemes for viscous and inviscid conservation laws with rough coefficients". In: ***Mathematical Modelling and Numerical Analysis*** 35.2 (2001), pp. 239–269. DOI: 10.1051/m2an:2001114.
