# Supplementary material for: Including population and environmental dynamic heterogeneities in continuum models of collective behaviour with applications to locust foraging and group structure
Source: PLoS Comput Biol. 2025 Apr 15;21(4):e1011469. doi: 10.1371/journal.pcbi.1011469 (PMC11999712; doi:10.1371/journal.pcbi.1011469)
Supplement: S4 Appendix — Derivation of parameters given in the simulation section. (PDF) [file pcbi.1011469.s004.pdf]

S4 Appendix: Parameter Estimation

Fillipe Georgiou[1], Camille Buhl[2], J.E.F. Green[3],
Bishnu Lamichhane[4] and Ngamta Thamwattana[4]
[1] Institute for Mathematical Innovation, University of Bath,
Bath, United Kingdom.
[2] School of Agriculture, Food and Wine, University of Adelaide,
Adelaide, Australia.
[3] School of Computer & Mathematical Sciences, University of Adelaide,
Adelaide, Australia.
[4] School of Information and Physical Sciences, University of Newcastle,
Callaghan, Australia.

February 24, 2025

For this section our full equation is given by:

$$\frac{\partial \rho}{\partial t} + \nabla \cdot (\boldsymbol{v}_x \rho) + \nabla_{\boldsymbol{n}} \cdot (\boldsymbol{v}_n \rho) = D \nabla \cdot \left[f_l(\boldsymbol{n}, E) \nabla \rho \right], \tag{1}$$

with

$$\boldsymbol{v}_x = -\nabla (f_n(\boldsymbol{n}, E) Q * \bar{\rho}) - D \left[\nabla f_l(\boldsymbol{n}, E) + \gamma f_l(\boldsymbol{n}, E) \nabla \left(\tau(\bar{\rho}) \right) \right], \tag{2}$$

and

$$\tau(\bar{\rho}) = \bar{\rho}^2, \tag{3}$$

$$Q(\boldsymbol{x}) = e^{-\frac{|x|}{r}}. \tag{4}$$

In addition, our gregarisation state equations are,

$$\dot{n}_g = \left(f(\bar{\rho}) - k n_g \right), \tag{5}$$

$$f(\bar{\rho}) = \frac{\delta \left(\frac{\bar{\rho}}{\kappa} \right)^2}{1 + \left(\frac{\bar{\rho}}{\kappa} \right)^2}, \tag{6}$$

and

$$f_n = f_{ng} = A \left(1 - \frac{2}{1 + e^{-15(n_g - 0.5)}} \right), \tag{7}$$

where A is the maximum strength of the interaction. Finally, for the state of hunger we have,

$$\dot{n}_h = \lambda(n_h) c(\boldsymbol{x}, t) - \nu n_h, \tag{8}$$

where $\lambda(n_h)$ describes how fast the locusts eat based on hunger, and ν is the energy lost due to metabolism. For the effect on movement we have

$$f_{lh} = 1.5 - n_h, \tag{9}$$

and for consumption we have

$$\lambda(n_h) = \eta_l (2 - n_h) \tag{10}$$

$$\psi(n_h) = \eta_f (2 - n_h) \tag{11}$$

1 Parameter Estimations

1.1 Estimating movement parameters

For our non-local movement parameters we begin by noting that the locust sensing radius is approximately 14 cm giving $r = 0.14$ [5, 8]. Then to find A we follow Topaz et. al. [8] exactly, in that the speed of a locust alone varies between 72-216 mhr^1 and in a group is 144-216 mhr^1 [2]. We also take the average maximum locust density as ≈ 1000 locusts m^2 [5] which in one dimension becomes $\rho_{\text{amb}} = 10\sqrt{10}$ locusts m.

We then imagine a hypothetical semi-infinite density field

$$\rho(x) = \rho_{\text{amb}}\mathcal{H}(x),$$

where $\mathcal{H}(x)$ is the Heaviside function. Placing a hypothetical locust at the edge of this group we can calculate its velocity as

$$v = Af_n(\boldsymbol{n}, 0)\left[-\nabla Q * \rho_{\text{amb}}\mathcal{H}(x)\right]_0, \tag{12}$$

$$v = Af_n(\boldsymbol{n}, 0)\left[\int_0^\infty \frac{1}{r}e^{-\frac{x}{r}}\rho_{\text{amb}}dx\right], \tag{13}$$

$$v = Af_n(\boldsymbol{n}, 0)\rho_{\text{amb}}. \tag{14}$$

We then assume that for a fully solitarious locust would move away from a maximally dense group at its top speed (and a fully gregarious locust to move towards the group at top speed), from this we find that $A \approx 6.83$.

For our local movement parameters we begin by assuming that there is very little purely random movement and set $D = 0.01$. This assumption allows the model to more accurately approximate compact supports (a property of swarms) [3] and brings the model more in line with previous studies that did not include linear diffusion or only included it to ensure numerical stability [6, 8]. Again, we take the average maximum locust density as $10\sqrt{10}$ locusts m [5]. Then, assuming $n_h = 0.5$ and $n_g = 1$ we can find $\gamma = 4.5291$ using (**??**).

1.2 Estimating gregarisation parameters

From (5), for a fixed $\bar{\rho}$ we can solve explicitly for $n_g(t)$ as

$$n_g(t) = \frac{f(\bar{\rho})}{k} + c_0e^{-kt}, \tag{15}$$

then we can find k by assuming $n_g(0) = 1$, $n_g(t^*) = 0.05$ and $f(\bar{\rho}) = 0$, i.e. a gregarious locust fully solitarises (within 5% as our functions are asymptotic) after time, t^*. This gives

$$k = \frac{-ln(0.05)}{t^*},$$

and letting $t^* = 3$ we get $k = 0.9986$. Next we can find δ by letting $n_g(0) = 0$, $n_g(t^*) = 0.95$ and $f(\bar{\rho}) = \delta$. i.e. a solitarious locust will fully gregarise (within 5%) at the maximum rate of gregarisation after time, t^*. This gives

$$\delta = \frac{k}{\frac{1}{0.95} - e^{-kt^*}}. \tag{16}$$

If again we let $t^* = 3$ (corresponding to symmetric gregarisation) we get $\delta = 0.9960$. Finally, to find κ, we look at locust density prior to the onset of collective behaviour. We begin by setting $\dot{n}_g = 0$, finding that

$$\kappa = \bar{\rho}^*\sqrt{\frac{1 - \frac{kn_g^*}{\delta}}{\frac{kn^*}{\delta}}}, \tag{17}$$

where $\bar{\rho}^*$ is the lower limit of collective behaviour (≈ 5 from [4]) and n_g^* is state at which the stability condition from (**??**) is first satisfied. We can then find,

$$n_g^* = \frac{1}{15}\ln\left(\frac{-2}{\frac{\bar{\rho}^* D\gamma + \frac{D}{\bar{\rho}^*}}{2Ar} - 1} - 1\right) + 0.5, \tag{18}$$

which gives $n_g^* = 0.5472$, which in turn gives $\kappa = 4.5351$. We can then see gregariousness in time at different locust densities in Figure 1, with the maximum density is the limit as $\bar{\rho} \to \infty$ of $f(\bar{\rho})$. This behaviour is comparable to that depicted in Figure 1C in [1] for gregariousness compared with treatment time.

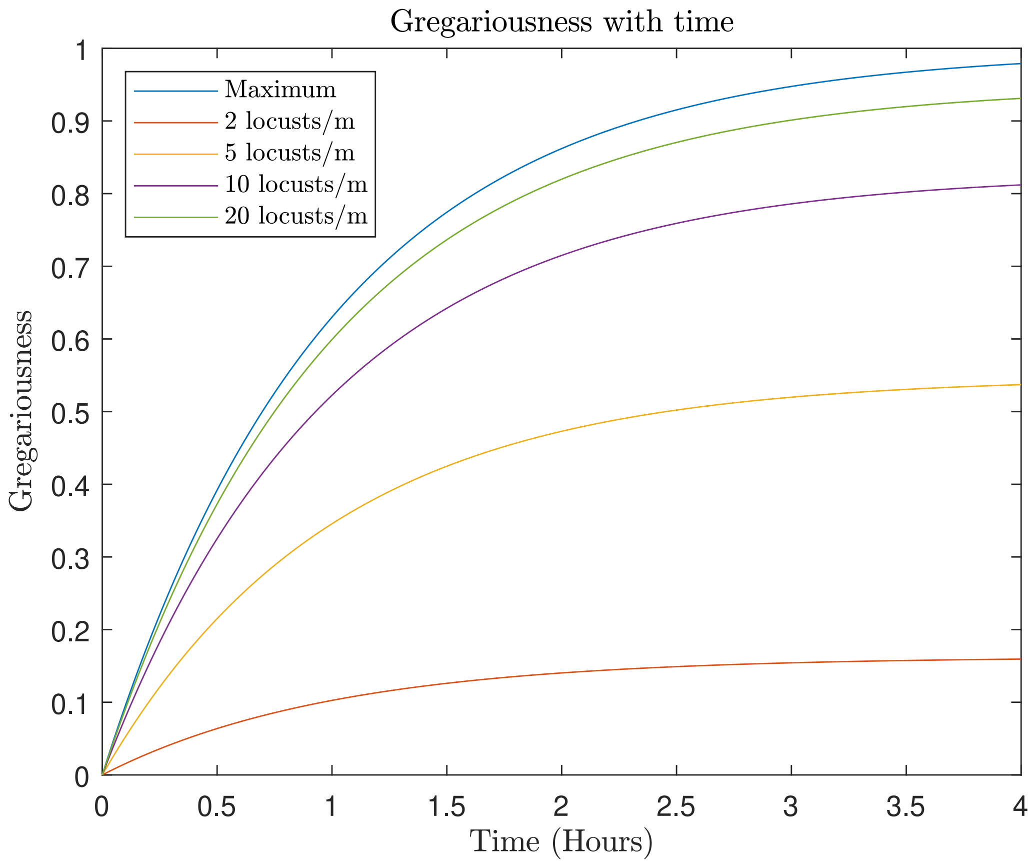

Figure 1: **Gregarisation in time.** Estimates of time required for a solitarious locust to reach various levels of gregarisation at different locust densities. The maximum density is the limit as $\bar{\rho} \to \infty$ of $f(\bar{\rho})$.

1.3 Hunger and food parameters

From (8) by assuming there is no food consumption, we get

$$n_h(t) = n_0 e^{-\nu t},$$

where n_0 is our starting satiation. Then, if it takes some time, t^*, for the satiation level to go from 1 to 0.05, we can calculate ν as

$$\nu = \frac{-\ln(0.05)}{t^*}. \tag{19}$$

Based on [7], after 1 day of starvation locusts reached their maximum amount of locomotion. If we let this be 12 hours of activity that results in $\nu = 0.2496$. We then estimate $\lambda(n)$, first by letting $\lambda(n)$ be linear in n, i.e. $\lambda(n) = an + b$ and our food be constant in time and space, we find

$$n_h(t) = \left(n_{\min} + \frac{bc}{ac-\nu}\right) e^{-(ac-\nu)t} - \frac{bc}{ac-\nu}.$$

So if we assume a locust stays completely satiated after eating for 80 minutes in a 5 hour period [7] (i.e. $an + b = 3.75\nu$) and a hungry locust eats twice as much as a satiated one we get

$$\lambda(\boldsymbol{n}) = 0.9362(2 - n_h). \tag{20}$$

Finally, for $\psi(\boldsymbol{n})$, we re-dimensionalise the parameter κ from our previous study [6], around $n_h = 0.5$ with the same linear relationship between hunger and consumption to get

$$\psi(\boldsymbol{n}) = 0.0384(2 - n_h).$$

References

[1] Michael L. Anstey et al. "Serotonin Mediates Behavioral Gregarization Underlying Swarm Formation in Desert Locusts". In: ***Science*** 323.5914 (Jan. 2009), pp. 627–630. DOI: 10.1126/science.1165939.

[2] S. Bazazi et al. "Nutritional state and collective motion: from individuals to mass migration". In: ***Proceedings of the Royal Society B: Biological Sciences*** 278.1704 (Feb. 2011), pp. 356–363. DOI: 10.1098/rspb.2010.1447.

[3] A. J. Bernoff and C. M. Topaz. "Biological Aggregation Driven by Social and Environmental Factors: A Nonlocal Model and Its Degenerate Cahn–Hilliard Approximation". In: ***SIAM Journal on Applied Dynamical Systems*** 15.3 (Jan. 2016), pp. 1528–1562. DOI: 10.1137/15M1031151.

[4] C. Buhl et al. "From Disorder to Order in Marching Locusts". In: ***Science*** 312.5778 (June 2006), pp. 1402–1406. ISSN: 0036-8075, 1095-9203. DOI: 10.1126/science.1125142.

[5] C. Buhl et al. "Group structure in locust migratory bands". In: ***Behavioral Ecology and Sociobiology*** 65.2 (Feb. 2011), pp. 265–273. ISSN: 1432-0762. DOI: 10.1007/s00265-010-1041-x.

[6] Fillipe Georgiou et al. "Modelling locust foraging: How and why food affects group formation". In: ***PLOS Computational Biology*** 17.7 (July 2021), e1008353. ISSN: 1553-7358. DOI: 10.1371/journal.pcbi.1008353.

[7] David Raubenheimer and Gerd Gäde. "Separating food and water deprivation in locusts: effects on the patterns of consumption, locomotion and growth". en. In: ***Physiological Entomology*** 21.1 (1996), pp. 76–84. ISSN: 1365-3032. DOI: 10.1111/j.1365-3032.1996.tb00838.x.

[8] C. M. Topaz et al. "Locust Dynamics: Behavioral Phase Change and Swarming". In: ***PLOS Computational Biology*** 8.8 (Aug. 2012), e1002642. ISSN: 1553-7358. DOI: 10.1371/journal.pcbi.1002642.
