# Supplementary material for: Including population and environmental dynamic heterogeneities in continuum models of collective behaviour with applications to locust foraging and group structure
Source: PLoS Comput Biol. 2025 Apr 15;21(4):e1011469. doi: 10.1371/journal.pcbi.1011469 (PMC11999712; doi:10.1371/journal.pcbi.1011469)
Supplement: Attachment — Submitted filename: PlosCompBio Review.pdf [file pcbi.1011469.s005.pdf]

The submitted manuscript addresses the formation of aggregates by a population of living organisms. The model uses an standard approach where movement fluxes are calculated accounting for the fact that perception is spatially-extended. As a consequence, movement rates are non-local, i.e. being a function of the density within a neighbourhood.
The main contribution of the work, in terms of modelling, is the introduction of (individual-level) internal states, such as the level of hunger, for instance. In their model, the typical partial/integro differential equation for a density rho, which has space and time, has an extra dimension (n). Therefore, the mathematical object they study is rho(x,t,n) which is the density of an individual at time t and location x in the state n.

The theoretical advances presented are motivated by experimental situations in which internal states depend on the position of individuals within aggregates. So, the model is providing a mathematical description for previously observed phenomena and is a contribution by itself. Overall, I find that the model and the math involved valuable for its specific literature. It is actually impressive the effort done in obtaining analytical results obtained. Nevertheless, after the revision of the current version of the results and text, I don’t think it has the potential to reach the broad audience of PlosCompBio. The main issue I see in the paper is the lack of straightforward take-home messages and I can’t identify if it is because of the presentation of the results, which might not be the most sharp and strongest choice, or due to the scope of the paper (being too technical). My comments below have in mind that the authors can actually say more about the results and provide valuable take-home messages in a future opportunity. If the authors are able to show that their contributions are more than a modelling increment (accounting for internal states), I think the paper should be reconsidered for PlosCompBio. Otherwise, it deserves a more specialised journal, perhaps more mathematical/technical, where the analytical calculation would be better received as they are.

It doesn’t take much time to understand that the model makes sense. In the introduction, we first understand that with discrete models we can’t do analytical calculation, so we should use continuous models. However, continuous models are limited, being one of these limitations the lack of internal states. So, the author's work contributes to a solution to this problem. But, we don’t know how much of a contribution it is. It is not clear what an interested reader would gain with it. What insights do the model reveal about locust behaviour? What about other species? This should be a straight answer in the abstract and introduction first paragraphs.

The text explains (quite well and easy to read) what happens when certain parameters change, i.e. ‘when this increases, that decreases’. The whole paper follows this report structure where you keep reading results, approximations, particular cases, variation of the model. While reading I wondered ‘where are we going with this?’. To solve this I think a change in the structure can be an option:

1) If the paper is indeed purely methodological, providing a framework to study the effects of the internal states in aggregation, I would much more strongly convey in the title/abstract/intro why someone should bother using your more complete but also more complicated approach. What is missing if we don’t use it? What prediction can be misled if we don't account for the role of internal states? And then connect to cases across species. The answer to some of these questions can be found in the

text but it is far from being central to the work in the current version. To be clear, I understand that the model reveals that X is more at the edge of the aggregate and Y is more at the center, but why is it important to know this? Did people know this before? Are you making any predictions or only validating experimental observations? These points are not clarified in the text.

2) If the paper actually brings a new result specific to locust I would recommend focusing on it. Being the case, the paper could be centralized around the locust problem which would be very helpful to keep the relevance and intuition about the results. Then, the authors can, in a proper space, say that the model is actually general and could be used to study different systems. This is a good example of takehome message that can be found in the Discussion Sec.: *"Our results with modelling hunger suggest a rather simple mechanism for this observed behaviour. In that, hunger increasing local dispersive movement drives the hungry individual to the edges of the group and has the additional effect of lowering the local maximum density. In addition, the most gregarious appear to be the most satiated, highlighting the advantage gregarisation offers when foraging [20, 21, 22, 24].".* This type of thing should guide the introduction. At this point it would also be useful to expand the "In addition" part going through what the refs say. Another good example is in the caption of Fig9: "We can see that hunger has led~~lead~~ to a greater loss of gregariousness and a reduction in the peak density thus leading to an increase in locust dispersal.". Although a good example, it is not perfectly clear the connection between height of the peak and dispersal is implicit here and in other parts of the text. Is it the reduction of the peak that leads to dispersal or the other way around: dispersal leads to the reduction of the peak?

   The issue regarding the structures becomes clear in one of the last sentences of the introduction: "It is these interactions of internal state that we aim to understand.". First, the "interaction" of internal states is a super confusing word here. It would be helpful to leave interaction for forces between individuals, here a different word would work better. Second, more importantly, the introduction ends without saying what the authors understood about aggregation after using the proposed model. So, the reader is left wondering what it is. And, in fact, this is only going to be more concretely stated in the discussion (see example above).

Other issues:

3) It is always hard to tune how much math is going to be displayed in an interdisciplinary journal. In the current case I think the authors stayed exactly in the middle. They show more math than the average (from my perspective) but it is not enough to understand where the equations are coming from. I would recommend hiding it more (appendix or SI) but extending their explanations. Some of the results like the small and large mass limits could be easily moved to the Appendice or SI (e.g. small and large mass limits). As you will see below I found that the terms of the equation were not sufficiently explained in the main text, making the equations very mysterious.

4) Is there a way of showing the impact of considering the internal states (relative to not considering it)? Maybe Fig 9 does the job but it is the last figure of the paper. I don't see any simple direct result where it is clear what changes when we go from the standard model (no internal states) to the proposed model. This would help address the takehome message issue.

5) Numerical experiments, i.e. Figs 5-9, should be motivated. All the subsections 'the effects of..' need to remember why the scenario considered is relevant and how it is connected to experimental observations. In the introduction these different scenarios are listed without much intention behind it. When making references to the relevant cases in the introduction (position-dependent internal state), the authors can directly warn the reader that they will be discussed later. And then, when addressing them in Sec 4, re-connect, remembering the reader about the reasons behind it.

6) Figs 5, 7 and 8 are too much work for the reader and they clash with the quality of the other figures. Suggestions: improve figures and use the text and visual aids to connect figs 4-5 and 6-7. For example, simulation trials are done over a specific line in the rho_amb-w space. A small detail: Fig 4 and 6 have weird vertical and horizontal lines (green) which seems to be a bug.

Minor issues:

Main Eqs. 2-3: What is E?

Eq 8: Minimisers? Where is this coming from? Which analytical method are you thinking about here? Add a brief description

Eq 3 is in 1D while Eq6 is in ND. Vx appears without definition. Also I wouldn't use the word velocity when talking about the flux through states, specifically when the work is about movement where an actual velocity exists.

The definition of vx in Eq 6 is completely mysterious. All the terms deserve to be mentioned but especially the \bar\rho^2 which is really noticeable and unusual.

No comments about the terms in Eq3, what each of them mean? Go through them. This will help to address issue #3

The abstract doesn't clarify the type of model that is going to be used. Is it an IBM? PDE? etc..

Abstract should be a single paragraph (?).

"These complex behaviours (...) lend themselves well to mathematical modelling" (Abstract) I don't understand what is the idea behind this sentence and it seems too vague for the abstract.

"However, this relationship might be more continuous than captured by that assumption"

Continuous at this point has been used for several things. I understood the sentence but it can be improved.

Do we need to see both panels in Figs 4 and 6? They are almost the same. Perhaps stick with the one that can be analytically calculated, or the most relevant one. If this makes sense the other panel can be present in the SI.

What does gregariousness mean in Fig. 9? Was it defined?
