# Supplementary material for: Including population and environmental dynamic heterogeneities in continuum models of collective behaviour with applications to locust foraging and group structure
Source: PLoS Comput Biol. 2025 Apr 15;21(4):e1011469. doi: 10.1371/journal.pcbi.1011469 (PMC11999712; doi:10.1371/journal.pcbi.1011469)
Supplement: Attachment — Submitted filename: ResponseLetter.pdf [file pcbi.1011469.s006.pdf]

Dear Ricardo Martinez-Garcia and James O'Dwyer,

Thank you for the kind words, and the consideration you have shown during the review process in general (in particular, accommodating the delay in re-submission). We have found both your and the reviewers' feedback extremely valuable. Please find attached to this letter the revised manuscript [PCOMPBIOL-D-23-01370] as well as responses to the reviewers comments.

Based on the suggestions we received, we have restructured the paper to focus on the locust example and moved the more mathematical results and derivations to the relevant appendices. We hope that by doing this we have produced a more coherent paper that is easier for, and more relevant to, the general audience. In addition, we have better highlighted some of the key results that can be obtained the heterogeneous model.

Thank you again,
Fillipe Georgiou

1 Editor

Dear Dr Georgiou,

Thank you very much for submitting your manuscript "Including organism and environmental heterogeneity in kinematic continuum models of collective behaviour with applications to locust foraging and group structure" for consideration at PLOS Computational Biology.
As with all papers reviewed by the journal, your manuscript was reviewed by members of the editorial board and by several independent reviewers. In light of the reviews (below this email), we would like to invite the resubmission of a significantly-revised version that takes into account the reviewers' comments.
I found your work very interesting and agree with all reviewers that introducing individual-level heterogeneity in continuous models is an important step forward in the collective behavior literature and in theoretical ecology broadly. However, I also agree with the Reviewers that, in its current form, the submission lacks focus on which are the relevant biological insights it provides. Also, given the broad readership that could be interested in understanding your framework to introduce individual-level heterogeneity in continuous models for different systems, I would encourage you to work hard on making the text more accessible for non-experts in locust biology and locust modeling. When preparing your revised submission, please respond appropriately to all Reviewer comments, but I encourage you to pay special attention to the two points above.
We cannot make any decision about publication until we have seen the revised manuscript and your response to the reviewers' comments. Your revised manuscript is also likely to be sent to reviewers for further evaluation.

When you are ready to resubmit, please upload the following:
1. A letter containing a detailed list of your responses to the review comments and a description of the changes you have made in the manuscript. Please note while forming your response, if your article is accepted, you may have the opportunity to make the peer review history publicly available. The record will include editor decision letters (with reviews) and your responses to reviewer comments. If eligible, we will contact you to opt in or out.
2. Two versions of the revised manuscript: one with either highlights or tracked changes denoting where the text has been changed; the other a clean version (uploaded as the manuscript file).

Important additional instructions are given below your reviewer comments.
Please prepare and submit your revised manuscript within 60 days. If you anticipate any delay, please let us know the expected resubmission date by replying to this email. Please note that revised manuscripts received after the 60-day due date may require evaluation and peer review similar to newly submitted manuscripts.
Thank you again for your submission. We hope that our editorial process has been constructive so far, and we welcome your feedback at any time. Please don't hesitate to contact us if you have any questions or

comments.

Sincerely,
Ricardo Martinez-Garcia
Academic Editor
PLOS Computational Biology

James O'Dwyer
Section Editor
PLOS Computational Biology

2 Reviewer 1

The submitted manuscript addresses the formation of aggregates by a population of living organisms. The model uses an standard approach where movement fluxes are calculated accounting for the fact that perception is spatially-extended. As a consequence, movement rates are non-local, i.e. being a function of the density within a neighbourhood. The main contribution of the work, in terms of modelling, is the introduction of (individual-level) internal states, such as the level of hunger, for instance. In their model, the typical partial/integro differential equation for a density rho, which has space and time, has an extra dimension (n). Therefore, the mathematical object they study is rho(x,t,n) which is the density of an individual at time t and location x in the state n.

The theoretical advances presented are motivated by experimental situations in which internal states depend on the position of individuals within aggregates. So, the model is providing a mathematical description for previously observed phenomena and is a contribution by itself. Overall, I find that the model and the math involved valuable for its specific literature. It is actually impressive the effort done in obtaining analytical results obtained. Nevertheless, after the revision of the current version of the results and text, I don't think it has the potential to reach the broad audience of PlosCompBio.

The main issue I see in the paper is the lack of straightforward take-home messages and I can't identify if it is because of the presentation of the results, which might not be the most sharp and strongest choice, or due to the scope of the paper (being too technical). My comments below have in mind that the authors can actually say more about the results and provide valuable take-home messages in a future opportunity. If the authors are able to show that their contributions are more than a modelling increment (accounting for internal states), I think the paper should be reconsidered for PlosCompBio. Otherwise, it deserves a more specialised journal, perhaps more mathematical/technical, where the analytical calculation would be better received as they are.

It doesn't take much time to understand that the model makes sense. In the introduction, we first understand that with discrete models we can't do analytical calculation, so we should use continuous models. However, continuous models are limited, being one of these limitations the lack of internal states. So, the author's work contributes to a solution to this problem. But, we don't know how much of a contribution it is. It is not clear what an interested reader would gain with it. What insights do the model reveal about locust behaviour? What about other species? This should be a straight answer in the abstract and introduction first paragraphs.

The text explains (quite well and easy to read) what happens when certain parameters change, i.e. 'when this increases, that decreases'. The whole paper follows this report structure where you keep reading results, approximations, particular cases, variation of the model. While reading I wondered 'where are we going with this?'. To solve this I think a change in the structure can be an option:

1) If the paper is indeed purely methodological, providing a framework to study the effects of the internal states in aggregation, I would much more strongly convey in the title/abstract/intro why someone should bother using your more complete but also more complicated approach. What is missing if we don't use it? What prediction can be misled if we don't account for the role of internal states? And then connect to cases

across species. The answer to some of these questions can be found in the text but it is far from being central to the work in the current version. To be clear, I understand that the model reveals that X is more at the edge of the aggregate and Y is more at the center, but why is it important to know this? Did people know this before? Are you making any predictions or only validating experimental observations? These points are not clarified in the text.

2) If the paper actually brings a new result specific to locust I would recommend focusing on it. Being the case, the paper could be centralized around the locust problem which would be very helpful to keep the relevance and intuition about the results. Then, the authors can, in a proper space, say that the model is actually general and could be used to study different systems. This is a good example of takehome message that can be found in the Discussion Sec.: "Our results with modelling hunger suggest a rather simple mechanism for this observed behaviour. In that, hunger increasing local dispersive movement drives the hungry individual to the edges of the group and has the additional effect of lowering the local maximum density. In addition, the most gregarious appear to be the most satiated, highlighting the advantage gregarisation offers when foraging [20, 21, 22, 24].". This type of thing should guide the introduction. At this point it would also be useful to expand the "In addition" part going through what the refs say. Another good example is in the caption of Fig9: "We can see that hunger has ledlead to a greater loss of gregariousness and a reduction in the peak density thus leading to an increase in locust dispersal.". Although a good example, it is not perfectly clear the connection between height of the peak and dispersal is implicit here and in other parts of the text. Is it the reduction of the peak that leads to dispersal or the other way around: dispersal leads to the reduction of the peak?

The issue regarding the structures becomes clear in one of the last sentences of the introduction: "It is these interactions of internal state that we aim to understand.". First, the "interaction" of internal states is a super confusing word here. It would be helpful to leave interaction for forces between individuals, here a different word would work better. Second, more importantly, the introduction ends without saying what the authors understood about aggregation after using the proposed model. So, the reader is left wondering what it is. And, in fact, this is only going to be more concretely stated in the discussion (see example above)

Thank you for thoughtful review of our paper. We certainly agree that it can be difficult to tune the amount of math to present in a paper of this nature, and we have thus taken your comments on board and refocused the paper onto the locust example with the more general mathematics and model derivation confined to the appendices. In addition by focusing on locusts we believe we have been able to tease out some key results as well as highlighting some of the extra information gained by including heterogeneity in the model. This has also resulted in a far cleaner paper.

2.1 Main issues

It is always hard to tune how much math is going to be displayed in an interdisciplinary journal. In the current case I think the authors stayed exactly in the middle. They show more math than the average (from my perspective) but it is not enough to understand where the equations are coming from. I would recommend hiding it more (appendix or SI) but extending their explanations. Some of the results like the small and large mass limits could be easily moved to the Appendice or SI (e.g. small and large mass limits). As you will see below I found that the terms of the equation were not sufficiently explained in the main text, making the equations very mysterious.

Thank you, based on your feedback we have restructured the paper to focus on locusts. In doing this we have moved a lot of the mathematics and general results to appendices and instead explain the key results within the context of locust behaviour.

Is there a way of showing the impact of considering the internal states (relative to not considering it)? Maybe Fig 9 does the job but it is the last figure of the paper. I don't see any simple direct result where it is clear what changes when we go from the standard model (no internal states) to the proposed model. This

would help address the takehome message issue.

We have restructured the numerical results section to show the need for internal state, by demonstrating that when only considering gregarisation the results are considerably different from when considering both gregarisation and hunger. We also highlight the nuance that is lost when the gregarisation dynamic is considered only binary rather than a spectrum.

Numerical experiments, i.e. Figs 5-9, should be motivated. All the subsections 'the effects of..' need to remember why the scenario considered is relevant and how it is connected to experimental observations. In the introduction these different scenarios are listed without much intention behind it. When making references to the relevant cases in the introduction (position-dependent internal state), the authors can directly warn the reader that they will be discussed later. And then, when addressing them in Sec 4, re-connect, remembering the reader about the reasons behind it.

Having restructured the paper, we believe that the coherence and flow has been improved. We hope that we now foreshadow, introduce, and tie each figure together better.

Figs 5, 7 and 8 are too much work for the reader and they clash with the quality of the other figures. Suggestions: improve figures and use the text and visual aids to connect figs 4-5 and 6-7. For example, simulation trials are done over a specific line in the rho_amb*-w space. A small detail: Fig 4 and 6 have weird vertical and horizontal lines (green) which seems to be a bug.*

We have combined figures 4 and 6 (and removed 5 and 8) so that the effect of the hunger dimension is more immediately obviouss. This also helps to introduce figure 7. In addition we have redone the figures so that the width changes are smoother. The weird lines in figures 4 and 6 appear to have been due to a export setting, that we believe we have now fixed. Finally, we have added in a colour bar with changing width to make the idea behind line thickness more visual.

2.2 Minor issues

Main Eqs. 2-3: What is E?

We have added further clarification around E, "Finally, we introduce a generic variable, E, to describe the environment. This might represent such environmental conditions as food density, sunlight, temperature, etc. In general, E may be a vector of values that change in space and time, and as a result of interactions with organisms."

Eq 8: Minimisers? Where is this coming from? Which analytical method are you thinking about here? Add a brief description

We have moved this portion of the derivation to the appendix, where we believe it makes sense within the broader context of gradient flows.

Eq 3 is in 1D while Eq6 is in ND. Vx appears without definition. Also I wouldn't use the word velocity when talking about the flux through states, specifically when the work is about movement where an actual velocity exists.

We have re-written the model section to be clearer and simpler, all derivations have been moved to the 1st appendix. In addition, we renamed Vn to a state-flux vector.

The definition of vx in Eq 6 is completely mysterious. All the terms deserve to be mentioned but especially

the $\bar{\rho}^2$ which is really noticeable and unusual.

We have added better explanations of the terms, for example ”To begin, we assume that $\tau(\bar{\rho})$, the local aspect of advective movement, is repulsive and proportional to the likelihood of collisions between locusts. We further assume that the probability of collision is independent of the state of the locusts and depends only on the local density. Using the law of mass action we get,”

No comments about the terms in Eq3, what each of them mean? Go through them. This will help to address issue 3

We have added explanation for each term

The abstract doesn't clarify the type of model that is going to be used. Is it an IBM? PDE? etc..

We have changed continuum to Partial Differential Equation in abstract

Abstract should be a single paragraph (?).

We have revised this to a single paragraph and removed extraneous sentences as well as clearer articulation of results.

"These complex behaviours (...) lend themselves well to mathematical modelling" (Abstract) I don't understand what is the idea behind this sentence and it seems too vague for the abstract.

Changed to ”These complex behaviours arise from simple individual and environmental interactions and are often modelled with the simplifying assumption of relative homogeneity of organisms. However, heterogeneity arising due to the internal state of individuals has an impact on these interactions and thus plays a role in group structure and dynamics."

"However, this relationship might be more continuous than captured by that assumption" Continuous at this point has been used for several things. I understood the sentence but it can be improved.

We have revised this to, ”However, observations of locust behaviour in experiments suggest that the degree of gregarisation might be depicted more accurately as a spectrum, rather than a binary solitarious/gregarious distinction ”

Do we need to see both panels in Figs 4 and 6? They are almost the same. Perhaps stick with the one that can be analytically calculated, or the most relevant one. If this makes sense the other panel can be present in the SI.

We have combined them into a single figure as part of the restructure of the numerical results section.

What does gregariousness mean in Fig. 9? Was it defined?

Changed to ”Mean level of gregarisation”

3 Reviewer 2

The manuscript addresses the formation of dense regions by moving organisms which are individually characterised by a state vector that can evolve in time depending on local conditions, somewhat similarly to some

chemical reactions between molecules. The authors devise a kinetic field theory of the reaction-advection-diffusion type. The effective force contributions are controlled by the current internal state and by a spatial distribution of environmental conditions such as food availability, temperature, etc. Some model features are discussed and numerical simulations performed, with applications to locust swarms. "Phase" separation into dense and dilute regions occurs if the overall density is within a certain range while a non-local attraction is sufficiently strong, a local diffusion is sufficiently low and a local repulsion is sufficiently low as well. For locusts, the authors find that more attractive (called gregarious) and less hungry individuals (where the level of gregariousness and of hunger define the internal state) will typically stay nearer the centre of the aggregates. For some conditions, agglomerates do not form even if initially attempted in the dynamics.

The motivation is certainly valid since combining two internal state dynamics is expected a priori to generate important modifications with respect to previous results by the same group in the same journal. Particularly interesting is the case where the initial conditions induce agglomeration but food is consumed faster than gregarization or hunger is maintained (as I interpreted it), therefore generating failed attempts to agglomerate. At the same time, the problem is clearly relevant to biology.

However, in my opinion, the manuscript has a large number of problems and the relevance of the final set of results is unclear despite the relevance of the model itself. I personally found that the text is poorly written. Many variables are not appropriately introduced, while modelling and (some important) parameter choices are not discussed, at least not in the main text. Having to go to the appendices multiple times is a problem. A large number of sentences are way more intricate than needed. Commas have been used as final periods (and vice versa) dozens of times, confusing the flow. Importantly, the rationale behind many modelling choices and results are not attempted to be explained heuristically, but rather just given, leaving the ultimate interpretation job to the reader. Also, I feel like the paper would have been much clearer if it started with the locust example straight away. Finally, the take-home novelty would be easier to grasp if the authors stated more clearly from the outset that the new ingredient with respect to their previous model is specifically the hunger dynamics.

Regarding the relevance of the results, although there are certainly a few insights which are significantly easier to obtain through the simulations, many others can be too easily anticipated only from stating the chosen model. There are not many quantitative results that can be used to obtain new unanticipated insights (the linear stability analysis is poorly performed; more details below). In fact, many insights highlighted by the authors are the same as in many other phase separation problems. In summary, although I do believe there are important insights, they get muddled in the middle of many repeated trivial results. On top of that, it is not completely clear which of the insights/results are actually new. By introducing hunger dynamics, individuals that get satiated move less and thus have a higher tendency to agglomerate and this can further induce agglomeration. Is that all? I think there is a bit more, but the punch line or take-home message is not clear at all, particularly in the abstract.

Therefore, I would recommend the text to undergo at least major revision. Below I raise many particular points, issues and suggestions, to try and help the authors with the process.

Thank you for the valuable feedback, we have hopefully addressed all your concerns. We think that by focusing entirely on locust behaviours (and relegating the general results to the appendices) we have made a much more coherent paper. In addition, we have been able to better present our results with relevance to the non mathematical community.

3.1 Individual Issues

Regarding the title/abstract, I'd understand it faster if it said "population" heterogeneity. The authors may also want to consider adding some word like "dynamic" or "time-evolving", so that it says "population and environmental dynamic heterogeneity".

How does "Including dynamic heterogeneity in continuum models of collective behaviour with applications to locust foraging and group structure" sound?

When the authors say "complex behaviors arise from simple ... interactions", they may want to say "relatively simple".

added "relatively"

For the abstract, authors may want to explain that gregarious means more sociable.

Added "(in a more sociable state)" after the first use of the word gregarious in the abstract.

Sentences like "finding that density and formation is most affected by the ratio of attractive to dispersive interactions." sound too obvious. Perhaps the authors can drop the word "finding" since this kind of behavior is old news and widespread in many models in the literature.

Removed these results to focus entirely on locusts.

First order SPPs can also capture alignment and pursuit. This division seems artificial. The authors can just say that inertia can be ignored for many animal movement problems.

Removed mention of the division, we just give some examples of their use.

All equations should be numbered. For the first equation (not numbered), the authors could just explain the mathematics of their modelling of the non-local contribution to the velocity as a convolution of a function (which, I don't know why, is expressed in terms of the grad of another function Q). Something like "We assume that each individual decides on its velocity based on a weighted average of the organism density in a neighbourhood around it, with closer individuals having higher weight." should suffice.

We have added a heuristic derivation to the appendix, and tidied up the model introduction section to hopefully make it clearer and easier to read.

There are sentences which are identical to those of Ref. [22].

Reworded introduction

Regarding the sentence "this relationship might be more continuous", only later I understood that "relationship" here meant the level of gregariousness.

Changed to "However, the degree of gregarisation might be more accurately depicted as a spectrum than a binary solitarious/gregarious"

When "environment" is first mentioned, one should already give the food availability example. It remained ambiguous before that.

Added extra details on what E might be to the model introduction section

For the term with ρ^2, the authors could explain that this corresponds to a flux velocity proportional to the negative of the gradient of ρ^2 since individuals want to move away from where collisions are most likely, like a repulsive autochemotaxis. The kind of explanation is necessary (I needed to look at other references). Besides, with that, no new microscopic derivation would be needed.

Added more explanation for each term, for ρ^2 specifically, "To begin, we assume that $\tau(\bar{\rho})$, the local aspect of advective movement, is repulsive and proportional to the likelihood of collisions between locusts. We

further assume that the probability of collision is independent of the state of the locusts and depends only on the local density. Using the law of mass action we get,”

Comparing the first unnumbered equation and Eq. (4), it seems that the gradient is taken after convolution only in the second case. Which is which and why?

Rewrote the first unnumbered equation to avoid confusion. Taking the gradient of a completed convolution is equivalent to taking the gradient of either term before performing the convolution.

Combining Eq. (6) and the unnumbered equation just below it, there seems to be a repeated term.

Terribly sorry but I don’t see it, combining the two equations recovers the sum of the flux terms. The additional $\nabla fl(n, E)$ arises due to creation/extraction of the linear diffusion term from the local flux.

For section 3, the authors should say in its title that it applies only to the simpler case without their dynamic heterogeneity.

Added caveats at the start of the section and note that it is valid for situations where the timescale of movement is far shorter than the timescale of state change.

In ”we will approximate the support of an aggregation”, the authors should explain the new capital omega (different than the omega prime) more carefully.

Changed to ”is infinite due to the linear diffusion the bulk of the mass is contained as a series of aggregations, we will approximate the finite support of a single aggregation as Ω” in the appendix, dropped entirely from the main text.

The sentence ”Finally, any change in state that increases. . . ” is covered by the ”vice versa” just before, right? If so, eliminate the repetition.

Great point, repetition removed.

Explain that function B in (14) is a special function so we don’t need to go to the appendix to check whether it is a new thing not defined before.

Added ”where B is the β-function (for definition see [], page 207)”

When the authors say ”the dotted lines represent the minimum estimates”, explain why the minimum is taken.

Added ”We use the minimum estimate as the small mass limit prediction is lower in the region where M is small, but as the small mass limit increases with M we would assume the results approach the constant large mass limit after the estimates cross over.”

For the 98% analysis, other percentages perhaps give somewhat different results. Is this value the one that gives the best comparison? Perhaps the authors could comment on that. In any case, I understand the idea here and that no big difference should arise for other percentages. No big deal.

Added that it was chosen arbitrarily.

Whenn_2 *is introduced in section 3.1.3, it seems the authors are calling* n_2 *the lever of hunger and thus*

more hunger means less diffusion. Is it not the level of satiation? This becomes even more confusing when n_2 becomes denoted by n_h below.

We have restructured the paper to introduce locusts and states earlier in the piece, this will hopefully avoid this problem.

Regarding the notation of some functions f_n or f_l, in some cases the E is kept in the list of dependent variables even when it is not there while in some other similar case it is not.

Within the main text we have changed E to c, we use E in the appendicies.

The modelling choices for f_n, f_l, f_c, etc. have to be justified heuristically. For instance, for the easiest one, a sentence like this would suffice: "Here we assume that the hungrier individuals decide to move more, which can be understood as a mechanism to find food quicker." or similar. Other cases are less obvious and should be discussed. These choices directly control final results. In fact, many final results can already be anticipated from these choices. They need to be justified. Just saying that a previous paper used it is not enough. Give at least a taste of why it is chosen like that.

Rewrote the model introduction section to include more explanation for functional choices. In addition, we give more detail around all of the parameter estimates.

Title and caption title of Fig. 2 do not seem accurate.

Changed to "Individual small and large mass simulations with estimations. Simulations of small ($M = 0.48$) and large $M = 96.17$ mass limits (left and right plots respectively). Here, $n_g = 0.95$, and $n_h = 0.15$, 0.55, and 0.95. In the plots, the vertical dotted lines are the estimates of the support, the horizontal dotted lines are the estimates of the maximum density, and the solid lines are the simulation results."

rho_c is introduced without definition in the main text. The same for the Fourier transform of Q.

We now introduce ρ_c and have removed the reference to the Fourier transform of Q.

For the two states analysis, s and g mean solitarious and gregarious. This should be said explicitly.

Changed to say: We can also consider two distinct sub-populations, a more gregarious state $\boldsymbol{n}_G$ and more solitarious state $\boldsymbol{n}_S$ with

$$\frac{f_l(\boldsymbol{n}_S, E)}{f_n(\boldsymbol{n}_S, E)} > \frac{f_l(\boldsymbol{n}_G, E)}{f_n(\boldsymbol{n}_G, E)}$$

(i.e. the ratio of non-local to local forces for $\boldsymbol{n}_G$ is less repulsive than for $\boldsymbol{n}_S$, or even attractive). While we have called our populations solitarious and gregarious the locusts don't need to be specifically in those states. For example, we could consider two solitarious populations with one being slightly less solitarious.

Regard using B below (20), notice that B was already used for the special function.

Changed to A_n and A_l.

In (20), phi_g bar is different than phi_g star. Explain the difference.

Restructured equations, and added ϕ^* *represents the critical gregarious fraction where aggregations begin to form (i.e. if the current gregarious fraction is greater than the critical fraction, then an aggregation is likely*

to form).

F_c, lambda(n) and psi(n) are introduced without definition in the main text.

Added definitions

Fig. 3 is not referred to in the main text. It just appears. By the way, is the y axis n_g? Please say it. Also give the number for the curve called "maximum" so that it can be compared to the others.

Moved to only be present in the appendices, and added The maximum density is the limit as $\bar{\rho} \to$ inf of $f(\bar{\rho})$.

The quantitative analytic results from the linear stability analysis (for instance, the transition values) are not compared to simulations, right?

They are currently not

Regarding (19), notice that Q also appears in the denominator. So taking only k=0 is not enough to identify the maximum lambda, right?

Thank you for pointing that out, we have added an additional portion to the analytic results showing that in the attractive region for gregariousness k=0 minimises the phi_g function. We also have found some further interesting results if $f_l(\boldsymbol{n}_S, E)f_n(\boldsymbol{n}_G, E) > f_l(\boldsymbol{n}_G, E)f_n(\boldsymbol{n}_S, E)$ we need to set k-¿infinity and our inequality only depends on the diffusivity (however this wont lead to aggregations).

What is the difference between rho_amb and $rho_c/2$?

Mathematically they are the same thing, we wanted to differentiate the ambient locust density in the numerical simulations from the constant one in the analytic results to avoid confusion.

After (37), explain that this shape is like a patch/plateau of food concentration surrounded by no food

Added "This corresponds to an isolated food patch surrounded by empty space."

The sentence "food decreases the required density... as opposed to optimal width" was very confusing.

Changed to "increasingly narrow food footprints decrease the required density for group formation"

In "Figure 5 we can see both" there is no "both" there.

Restructured the results and removed that paragraph.

I do not think using thickness of the line as a plotting technique was a good choice. It is not sufficiently clear.

We have tweaked the plotting script so it looks neater, we will consider some different approaches if it is still unclear. Perhaps opacity could be used?

Explain heuristically the re-emergence of optimal width effect. I believe this kind of insight is what will make the paper valuable.

Added: We can then hypothesise that this optimal food width is likely driven by repulsion, and with hunger being a key component of repulsion is a likely driver.

There are some typos. Please use a checker.

Changed latex program, hopefully overleaf has caught them.

In the discussions, rho bar is used as a parameter when it is actually a variable (the authors probably meant rho_c *or* $rho_i nf$*).*

Change to "steady-state density" to avoid the use of symbols.

I would not use the word "support". This is unnecessary mathematical jargon for the bio community. Just say the size of the patch or agglomerate or similar.

Added "we will label the footprint of this aggregation as the support, Ω" to the analytic results section, and reworded the discussion to avoid the word.

There are long parts in the appendices which are almost identical repetitions from other parts.

The restructure of the paper has hopefully avoided this duplication.

The result described in "When the food is too narrow there is attempted" is the most insightful. I would suggest putting a focus on this.

Added greater focus on this to the results section.

4 Reviewer 3

This study takes a class of previously explored locust models (by the authors and many others) and adds an internal state variable to model heterogeneity with an eye on locust biology (notably modeling hunger and degree of gregariousness). The models here are well motivated but by adding a state space to these models one observes an explosion of parameters and unknown functions and essentially increases the dimensionality of independent variables in the model significantly increasing the complexity. The models here are challenging (dare I say approaching unwieldy) but the authors skillfully finesse some insight from them. This reviewer believes that the authors should be praised for exploring this territory and believes that studies incorporating heterogeneity akin to what is done here are a path forward in continuum modeling of swarms. The writing of the paper assumes (in this reviewer's opinion) a significant familiarity with past work in this field which limits its accessibility. It could use a thorough editing with an eye on readability for a broader audience. The comments below point out a number of things that struck this reviewer that could be elucidated.

Thank you for the review and kind words. We have refocused the paper onto the the locust example with the more general mathematics and model derivation confined to the appendices. We feel that less familiarity with the techniques is now required to understand the main text. In addition by focusing on locusts we believe we have been able to tease out some key results as well as highlighting some of the extra information gained by including heterogeneity in the model. This has also resulted in a far cleaner paper.

4.1 Issues

Line 42: ". . . a the locust . . . "

Removed the 'a'.

Lines 72-75: Could be written clearer and with more specificity: Line 74: ". . . . local interactions are repulsive. . . " What about gregarization? Isn't this a local interaction?

Added clarifying movement interactions

Line 75: Why does 4 call out "any state" but 3 does not? Also is the state of the target organism, or the influencing organism (or both) that matters/doesn't matter?

Great point, we have removed the reference to the state of the target organism from the 4 and added to 5; "... and not by those of the target".

Lines 78-84: At least some comment should be made here about continuous/discrete states. As an example many previous models have made solitary/gregarious a binary state. Is this allowed in this model or not? Also, a discrete model is introduced later on - could this be foreshadowed?

There is no reason you couldn't also include some kind of binary state in the form of kinetics (or multi population)

Line 80-81: Why not define Ω_n here as opposed to line 85? Also is there any difference between "state space" and "complete state domain".

We now introduce Ω_n in line 80. It was to distinguish between the space and the region that we were investigating, but we realise this confuses things thus we will only refer to the state space.

Line 87: The definition here of E is vague – is a vector of variables (food density, sunlight, etc)? Do they depend on space? time?

Changed to "... a generic term for the environment, E, that might represent such environmental conditions as food density, sunlight, temperature, etc. E may be a vector of values that is constant, or change in space and time, or due to interactions with organisms, etc."

Line 93: The use of "a movement" here is confusing (nothing is moving) – change or evolution might be clearer.

Changed to "We also allow for continuous change within the state space"

Line 94: There is a subtlety in the flux equation I'd encourage the authors to call out explicitly – this form means that the state of a locust evolves continuously (i.e. it doesn't jump between states) in time (assuming the fluxes are not infinite). I believe this is the intent of the author and it should be made clear here or earlier in the text.

Added "and that this state evolves continuously in time" to the state flux introduction.

Line 101: While D is a diffusion constant, gamma here most certainly is not. It is a advective flux mediated by the function $\tau(\bar{\rho})$ introduced in the appendix. I'd also say that $f_l(n, E)$ is a mobility. In any case, the authors should spend a few lines explaining biologically what these terms here. I think the first term is diffusion of organisms where the diffusion constant is mediated by the environment and the organism state and the second term is an advective flux along gradients away from concentration densities – but the authors can surely do a better job of giving intuition about what is going on here. Along these lines talking about units for the constants may be useful here.

We have introduced clarifying statements in what each term means/relates to after stating the final equation.

Line 106: Might it be better to say the social potential is a function of distance between organisms and independent of location, state and time. The use of the word "space" here is ambiguous.

Changed to "we assume that the movement of each organism is a velocity based the distance weighted average of the surrounding organism density. However, now we assume that the weighting is only a function of distance (i.e. its constant in state and time, and independent of the organisms location) and the strength (and direction) is mediated by both the internal state and the environment."

Line 106 and 109: It seems to me that only the strength of the social potential is mediated by the internal state and the environment. I'm not even sure what is meant by "non-local force" here.

Reworded non-local force to social potential

Line 110: I'd encourage the authors not to use the term "velocity" here or to explain it is an analogy here. Even before lunch, I would be unlikely to say my hunger is increasing at 30 meters/second.

That would be a high speed hunger, we have renamed v_n to state vector field.

Line 133: The manuscript says "all the organisms are in the same unchanging state" the description of which is rather vague (I think unchanging here means relative to time but even that is unclear to me) – I think the authors want an N-dimensional delta function here for the state vector, but part of the problem goes back to line 113 because we have no idea if the state could evolve in time. I believe the authors want to say something like "suppose the state vector is constant and that $v_n = 0$ so that the state remains constant for all time." Ideally there would be a topic sentence earlier in section 3.1 that tells the reader that this approximation is being made. This also will need to be made consistent in Appendix B

We think that the results should remain valid in the case where the change in state is far slower than the movement speed of the locusts/organisms. I.e. of the order ϵ^2 in the case of the linear stability analysis. We would ideally like to work with a state delta function but we have used the approximation of a very small region of the state space where f_l and f_n could be considered constant over to try to avoid any issues with singularities.

Line 135-136: The evolution equation here has a long history and the author should give a few references. Notably as the equation is introduced here they should note that $\bar{\rho} = 0$ OR the variation in energy (after 137) is zero. Considering the large mass solutions that are discussed soon after have compact support this discussion should foreshadow this. Also, strictly speaking the equation after 137 only defines a steady state (not necessarily a minimizer).

Added reference to the Gradient flows text [**Ambrosio˙Gigli˙Savare˙2008**] and noted that the variation in energy will be zero where there are organisms, or the organisms density will be zero.

Line 206: Presumably the subscripts g and s are nods to gregarious and solitarious but this is not spelled out for the reader – a topic sentence about this at the beginning of 3.2.2 would be useful.

Added a topic sentence to the linear stability analysis section: "We can also consider two distinct sub-populations, a more gregarious state $\boldsymbol{n}_G$ and more solitarious state $\boldsymbol{n}_S$ with

$$\frac{f_l(\boldsymbol{n}_S, c)}{f_n(\boldsymbol{n}_S, c)} > \frac{f_l(\boldsymbol{n}_G, c)}{f_n(\boldsymbol{n}_G, c)}$$

(i.e. the ratio of non-local to local forces for $\boldsymbol{n}_G$ is less repulsive than for $\boldsymbol{n}_S$, or even attractive). While we have called our populations solitarious and gregarious the locusts don't need to be specifically in those states. For example, we could consider two solitarious populations with one being slightly less solitarious."

Line 239: The term "separable" here should be defined mathematically and described biologically.

Added: A full list of functions derived in this section can be found in Table 2. Finally, for simplicity we assume that the individual state and environmental components of f_n and f_l are separable. Mathematically this is,

$$f_n = f_{ng}, \text{ and } f_l = f_{lh} \cdot f_c.$$

Biologically, this means that the long range interactions are only affected by the gregarisation, that locusts stop at food at the same rate regardless of hunger (they simply change their rate of consumption), and that the extra dispersion due to hunger is not affected by the amount of food present. These assumptions may be a bit simplistic but they do allow us to easily derive parameters from the available data.

Section 4: The authors should explain why the particular range of locust densities is used and why figure 4 and 6 have that density decreasing with vertical y (the authors may make this choice if they have a good reason but they should warn the reader).

Added: We begin by considering ambient locust densities around the expected onset of group behaviour ($\approx \rho_{\text{amb}} = 5$) from $\rho_{\text{amb}} = 4.5$ to $\rho_{\text{amb}} = 5.2$

Additional corrections

During the course of the revision we found that the simulation results with a food footprint $\omega = 2.5$ contained numerical error due to the high gradients at the food boundary, we have removed these results. We believe the other numerical simulation results to still be correct.
