# Supplementary material for: Including population and environmental dynamic heterogeneities in continuum models of collective behaviour with applications to locust foraging and group structure
Source: PLoS Comput Biol. 2025 Apr 15;21(4):e1011469. doi: 10.1371/journal.pcbi.1011469 (PMC11999712; doi:10.1371/journal.pcbi.1011469)
Supplement: Attachment — Submitted filename: Notes_on_Paper_Template.pdf [file pcbi.1011469.s007.pdf]

Referee's report: Including dynamic population and environmental heterogeneity in continuum models of collective behaviour with applications to locust foraging and group structure

July 8, 2024

1 Summary

The authors have significantly restructured the paper, and in the opinion of this referee the new presentation is easier to read. As I said in my previous review, the problem being examined here is ambitious due to the complexity of the equations and the sheer number of parameters and effects included. The paper demonstrates that including hunger and gregariousness refines are understanding of the effects that govern group formation. The analytical stability and scaling results also present a nice scaffolding for intuition about the problem. There is also ample room for further exploration.

I've made a number of comments below - while they should be addressed they are mostly minor and hopefully will improve the accessibility of the manuscript.

Notes on the the governing equation

It is essential that the authors give a clear and coherent derivation of the governing equations. Let me start out by pointing out what I presume is an error:

- Line 126: In Equation (7) what might be called the mobility $f_n(n, E)$ is inside the gradient – the derivation in the Appendix (and physical intuition) suggests it should be outside the gradient. This is wrong in equation (10) and equation S1 (33) also.

A second item which it took me quite awhile to figure out is that:

- Near line 133 (main paper) at equation (9) and (10) and also Supplement S1 equation (33): The linear diffusion term in equation (9) and S1(33) splits the local linear diffusion term into a velocity that depends on the

gradient of the environmental term and a term that depends on the gradient of the density of locusts in a given state and puts one on each side of teh equation.

- Meanwhile the nonlinear diffusion term in equation (10) has a contribution to the velocity that depends on the gradient of the total density $[\nabla(\tau(\bar{\rho})]$.

The authors should exlain explicitly why/how these separations are made. It isn't clear to me why equations (9) & (10) are any better then equation (4), and while it is the authors choice they at least owe the reader an explanation in my humble opinion.

Finally a small item:

- In the supplemental material S1 between equation (22) and (33) the local fluxes go from one to two dimensions - while this is no worse than what is done in Painter and Sherratt [34] the authors should add a line of explanation.

When these items are addressed the authors should circle back to lines 86-93: I think what you are trying to do here is classify the interactions, both between organisms and between organisms and the environment, but the language is muddled. In particular, 5 contradicts 3 (where it says *regardless of internal state*) and 3 and 4 are not parallel. I think the main idea is that an organism can only sense the environment locally and that the density of other organisms both locally and non-locally (but not the state of other organisms). Another item is that the state of an organism changes continuously (it doesn't jump). Do the authors want to say something about how the environmental variables evolve here also?

Minor Comments

- Abstract/Author summary: The authors use terms like "optimal food" and "optimal food width" that are unclear. I think they mean something like patch size but they should strive for more clarity.
- Line 8: "only vary in one or two discrete ways," perhaps replace with "only assume a small number of discrete states" Using the term "vary" here suggests a continuous variable.
- Line 17: Delete "As previously mentioned"
- Line 23: "dependant" should be "dependent"
- Line 40: "presecribed" should be "prescribed"
- Line 76-77: "with all interactions mediated by the internal state of the organism" perhaps should be "where these interactions may be mediated by the internal state of the organism"

- Line 83: Perhaps insert the word “unit” before hypercube?
- Line 97: For equation (3) isn't mass conserved in this paper? Do you want to state that M is constant?
- Line 109: “around” should be “within”
- Line 114: Equation (6) seems to directly contradict the statement in point 3 on line 89 that says “regardless of their internal state”
- Line 122: The term “average” here is misleading as it suggests you divide by the total mass. Equation (7) suggests you are doing otherwise. I think you want something like “velocity is a distance-weighted sum”
- Line 143: The potential is called the ”Morse potential” and is worth name-checking for the reader.
- Line 153-154: I think the authors are saying that f_l is a product of a term depending solely on c and a term depending solely on n - if so, could one help the reader realize this by writing

  $$f_l(n, E) = f_c(c) * f_{lh}(n).$$

  You might point out that f_{lh} is related solely to hunger and therefore is $f_{lh}(n_h)$.
- Line 176 and equation (17)/Line 184 and equation (19): As written the reader has no idea of what f_{ng} is. I think you are trying to say something like:

  $$f_n(n, E) \equiv F(n_g) = ...$$

  but some more clarity would be appreciated.
- Equation 19: Can you insert a reference to where f_{lh} appears earlier in the text?
- Table 1: Perhaps add $f_l = f_c f_{lh}$ to this table?
- Lines 161-188: It isn't obvious to this reader that n_g and n_h are bounded above by 1 as is stated on line 80? Can you explain this? It might also be useful to note that they are bounded below by zero by the form of equation 15 and 18.
- Line 244-245: perhaps put both $M = ...$ in parentheses. Also in the caption to Figure 2.
- Line 256: Replace ”??” (Appendix reference)
